# Plant Nitrilase Homologues in Fungi: Phylogenetic and Functional Analysis with Focus on Nitrilases in *Trametes versicolor* and *Agaricus bisporus*

**DOI:** 10.3390/molecules25173861

**Published:** 2020-08-25

**Authors:** Lenka Rucká, Natalia Kulik, Petr Novotný, Anastasia Sedova, Lucie Petrásková, Romana Příhodová, Barbora Křístková, Petr Halada, Miroslav Pátek, Ludmila Martínková

**Affiliations:** 1 Laboratory of Modulation of Gene Expression, Institute of Microbiology, Czech Academy of Sciences, Vídeňská 1083, CZ-142 20 Prague, Czech Republic; rucka@biomed.cas.cz (L.R.); patek@biomed.cas.cz (M.P.); 2 Centre for Nanobiology and Structural Biology, Institute of Microbiology, Czech Academy of Sciences, Zamek 136, CZ-373 33 Nové Hrady, Czech Republic; kulik@nh.cas.cz; 3 Laboratory of Biotransformation, Institute of Microbiology, Czech Academy of Sciences, Vídeňská 1083, CZ-142 20 Prague, Czech Republic; petr.novotny@biomed.cas.cz (P.N.); anastasia.sedova@biomed.cas.cz (A.S.); petraskova@biomed.cas.cz (L.P.); romana.prihodova@biomed.cas.cz (R.P.); barbora.kristkova@biomed.cas.cz (B.K.); 4 Faculty of Biomedical Engineering, Czech Technical University in Prague, nam. Sítná 3105, CZ-272 01 Kladno, Czech Republic; 5 Laboratory of Structural Biology and Cell Signaling, BioCeV-Institute of Microbiology, Czech Academy of Sciences, Průmyslová 595, CZ-252 50 Vestec, Czech Republic; halada@biomed.cas.cz

**Keywords:** fungi, plant nitrilase homologues, β-cyano-L-alanine, arylaliphatic nitriles, fumaronitrile, substrate specificity, homology modeling, *Trametes versicolor*, *Agaricus bisporus*, plant-fungus interactions

## Abstract

Fungi contain many plant-nitrilase (NLase) homologues according to database searches. In this study, enzymes NitTv1 from *Trametes versicolor* and NitAb from *Agaricus bisporus* were purified and characterized as the representatives of this type of fungal NLase. Both enzymes were slightly more similar to NIT4 type than to NIT1/NIT2/NIT3 type of plant NLases in terms of their amino acid sequences. Expression of the synthetic genes in *Escherichia coli* Origami B (DE3) was induced with 0.02 mM isopropyl β-D-1-thiogalactopyranoside at 20 °C. Purification of NitTv1 and NitAb by cobalt affinity chromatography gave ca. 6.6 mg and 9.6 mg of protein per 100 mL of culture medium, respectively. Their activities were determined with 25 mM of nitriles in 50 mM Tris/HCl buffer, pH 8.0, at 30 °C. NitTv1 and NitAb transformed β-cyano-L-alanine (β-CA) with the highest specific activities (ca. 132 and 40 U mg^−1^, respectively) similar to plant NLase NIT4. β-CA was transformed into Asn and Asp as in NIT4 but at lower Asn:Asp ratios. The fungal NLases also exhibited significant activities for (aryl)aliphatic nitriles such as 3-phenylpropionitrile, cinnamonitrile and fumaronitrile (substrates of NLase NIT1). NitTv1 was more stable than NitAb (at pH 5–9 vs. pH 5–7). These NLases may participate in plant–fungus interactions by detoxifying plant nitriles and/or producing plant hormones. Their homology models elucidated the molecular interactions with various nitriles in their active sites.

## 1. Introduction

The nitrilase (NLase) superfamily proteins were examined for their metabolic roles (biotin recycling, pyrimidine catabolism, NAD synthesis, tumor suppression and acceleration of protein turnover) and their potential as biocatalysts (NLases, amidases, carbamoylases). The common feature of the NLase superfamily enzymes is the E-K-C catalytic triad [1,2]. Later, another E residue was found to be essential for the catalytic mechanism. Hence, the catalytic site was designated a tetrad [3]. The enzyme activities reside in the transformation of C-N groups such as nitrile, primary amide, secondary amide or carbamoyl [1,2].

The NLase-superfamily proteins are classified into 13 branches with different activities and substrate specificities. Branch 1 contains NLases (EC 3.5.5.-), which hydrolyze or hydrate the C≡N bond. The biocatalytic potential of NLases is significant due to their ability to transform dozens of nitriles into carboxylic acids or amides at ambient temperature and mild pH. Moreover, NLases are often stereo- and regioselective; their selectivities depend on both the enzyme and substrate. NLases are sensitive to agents interacting with their catalytically active C residue (e.g., *N*-ethylmaleinimide) [4] and, in some cases, also to high substrate concentrations [3,5].

New NLases were obtained by screening, (meta)genome mining or mutagenesis [5]. These enzymes exhibited a great variability in their substrate specificities, product composition (amide:acid ratio) and stereo- or regioselectivities. The main source of NLases acting on aromatic or (aryl)aliphatic nitriles was bacteria [3]. Currently, a number of NLases are also available commercially from Prozomix [6], Codexis [7] or Sigma [8].

Several plant NLases (EC 3.5.5.1) were also characterized and classified into NIT1/NIT2/NIT3 type or NIT4 type. The former acts on various (aryl)aliphatic nitriles, whereas the latter is highly specific for β-cyanoalanine (β-CA) [4,9,10,11]. Nitriles are formed during the breakdown of plant defense compounds, cyanogenic glycosides (CGG) or glucosinolates, on damage of the plant tissues by, e.g., herbivores or parasites. Both types of compounds are cleaved into carbohydrate and aglycon. The aglycons of CGG, 2-hydroxynitriles, decompose into aldehyde and the highly toxic HCN. The unstable aglycons released from glucosinolates also give rise to toxic products, among them phenylacetonitrile (PAN) or indole-3-acetonitrile (IAN) [12]. HCN can be also formed as a side product of the biosynthesis of the plant hormone ethylene (a key substance in plant signaling networks [13]). HCN reacts with L-cysteine to give β-CA [12]. This reaction is catalyzed by β-CA synthase (EC 4.4.1.9) and it is followed by the hydrolysis of β-CA with NLase NIT4 to give Asn, Asp and ammonia. Thus, NIT4 not only enables nitrile detoxication but also nitrogen recycling [11]. In addition, phenylacetic acid (PAA) and indole-3-acetic acid (IAA), which can be obtained by the hydrolysis of PAN and IAN, respectively, are plant hormones. Plants can produce them from glucosinolates as described above but also from amino acids via aldoximes and nitriles [12]. Nevertheless, it was proposed that NLases only serve to produce IAA in specific plants (*Brassicaceae*, *Poaceae*, *Musaceae*) and under specific conditions, such as sulfur deficiency [11].

In the past decade, the search for new NLases also encompassed fungi as their underexplored sources. Thus, over 15 NLases from *Ascomycota* were produced in *E. coli* and the majority of them were purified [14]. Both bacterial and fungal NLases were largely classified as aromatic NLases (substrates: aromatic nitriles) or arylacetoNLases (substrates: arylaliphatic nitriles). Fungi, mainly *Ascomycota*, are also a source of cyanide hydratases (CynHs), whose specific activities are the highest for HCN. CynHs are related to NLases with below 40% amino acid sequence identities (hereinafter only identities) [15,16,17].

In *Basidiomycota*, the first three NLases and a CynH were recently described by us [17,18]. They were overproduced in *E. coli* and their activities were examined using whole cells [18] except for the arylacetoNLase from *Auricularia delicata* [17], which was purified. This enzyme is unique in *Basidiomycota*, having no close homologues in this division [18].

One of these NLases, NitTv1 from *Trametes versicolor*, was similar to plant NLases with identities of up to >50%. The whole-cell activity assays indicated that its substrate was β-CA as in NIT4 but also some (aryl)aliphatic nitriles as in NIT1-NIT3 [18]. However, a more detailed biochemical characterization of the enzyme was required to compare its properties with those of plant NLases.

Therefore, NitTv1 was purified and biochemically characterized in this work. Based on this characterization, we proposed its possible roles in fungi and plant-fungus interactions. The other NLase examined in this study (NitAb) was from *Agaricus bisporus* var. *bisporus*. The enzyme is unique in *Basidiomycota* but has a number of homologues in *Ascomycota*. It exhibited a lower but significant identity to plant NLases (ca. 40% to NIT4). The two fungal NLases were compared with plant NLases in terms of their primary structures and catalytic properties. The catalytic potential of the new NLases was also estimated.

A homology model of NitTv1 was recently constructed and used for substrate docking [18]. Molecular modeling was also involved in this study to shed light on the interactions of substrates in the active sites of the new fungal NLases. The established in silico models will enable to predict, with some probability, further substrates of these NLases and their homologues.

## 2. Results

### 2.1. Analysis of Published Sequences

Previously, the published NLase sequences in division *Basidiomycota* (subdivision *Agaricomycotina*) were classified into two main clades. Members of clade 1 were similar to plant NLases with up to >50% identities, while those of clade 2 exhibited up to ca. 50% identities to “aromatic” NLases in *Ascomycota*. In addition, five CynHs were found [18].

NitTv1 (sequence XP_008032838.1) was selected as a representative of clade 1. The number of NLases classified in this clade was previously over 60 after excluding highly similar sequences [18]. An updated analysis of the GenBank [19] gave over 80 sequences with <99% identities to each other.

In this work, NitTv1 was purified and characterized along with NitAb (sequence XP_006462086.1) from *A. bisporus* var. *bisporus*. NitAb is outside clade 1 in the phylogenetic tree (Figure S1 in Supplementary Materials). It has a single close homologue in *Basidiomycota* (sequence XP_007327111.1 in *A. bisporus* var. *burnettii* with almost 99% identities), but >500 homologues with >50% identities in *Ascomycota*. Hence, it was selected as a representative of this group of NLases. The corresponding genes (designated *nitTv1* and *nitAb*) were optimized for *E. coli*. The optimized sequence of *nitTv1* was reported previously [18] and that of *nitAb* is shown in Figure S2 (Supplementary Materials).

**Figure 1:**
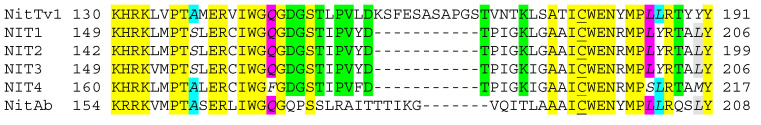
Multiple sequence alignment of the catalytic regions in plant nitrilases from *Arabidopsis thaliana* (NIT1–NIT4) and fungal nitrilases NitTv1 and NitAb. The catalytic C residue is underlined. Amino acid residues conserved in all sequences are in yellow. Those conserved in NitTv1 and plant NLases are in green. Amino acid residues conserved in the NIT1/NIT2/NIT3 type and different in NIT4 type are in italics. The residues identical in NIT1/NIT2/NIT3 and fungal NLases are in magenta and those identical in NIT4 and fungal NLase in blue. The residues identical in NIT1/NIT2/NIT3 and NitAb (but different in NitTv1) are in grey.

The identities between NitTv1, NitAb and the characterized plant NLases NIT1 (NP_001078234.1), NIT2 (NP_190016.1), NIT3 (NP_190018.1) and NIT4 (NP_197622.1) from *Arabidopsis thaliana* were determined (Table 1). NitTv1 exhibited a higher degree of identity to NIT4 (ca. 52%) than to NIT1-NIT3 (ca. 47–49%). NitTv1 was more similar to the plant NLases than NitAb (≈50% vs. ≈40% identities). The catalytic regions in plant NLases and their fungal homologues were also compared (Figure 1). The regions surrounding the catalytically active C residue (62 residues in NitTv1) were aligned.

A total of 23 residues (yellow in Figure 1) were the same in the fungal and plant NLases, between them the catalytic C and three residues downstream (WEN). This pattern only exhibits minor variations throughout NLases [3,18]. In addition, nine residues (green in Figure 1) were the same in NitTv1 and the plant NLases but different in NitAb. Five residues (italics in Figure 1) are the same in NIT1–NIT3 but different in NIT4. In fungal NLases, two of these residues were found to be the same as in NIT1-NIT3 (magenta in Figure 1) and two of them (blue) the same as in NIT4 (blue in Figure 1). The fifth one was the same in NIT1–NIT3 and NitAb but different in NitTv1 (grey in Figure 1). Hence, this analysis did not enable to judge on the classification of the fungal NLases as either NIT1–NIT3 or NIT4 type. Both fungal NLases have inserts which are not present in plant NLases. This region corresponds to loop HL2 in the homology model of these NLases (see Section 2.3 and Figure S3 in Supplementary Materials).

### 2.2. Overproduction and Characterization of Fungal Nitrilases

The expression of *nitTv1* and *nitAb* genes was performed in *E. coli* Origami B (DE3) at low isopropyl β-D-1-thiogalactopyranoside (IPTG) concentration (0.02 mM) and low temperature (20 °C) as in the previous study [18]. Unlike previously, however, the GroEL/ES chaperone was not induced. This was because the chaperone often co-purified with NLases and thus hampered their purification [20]. The cells were sonicated and the enzymes carrying *C*-terminal His_6_-tags were purified by cobalt affinity chromatography on TALON^®^ (see Figure S4 in Supplementary Materials for an overview of the enzyme preparation protocol) to give 6.62 ± 0.66 mg and 9.63 ± 0.33 mg of protein per 100 mL of culture medium. The identity of the proteins was confirmed by matrix assisted laser desorption/ionization—time-of-flight mass spectrometry (MALDI-TOF MS) analysis of their bands in SDS-PAGE. The sequence coverage for NitTv1 and NitAb was 71% and 63%, respectively (Figure S5 in Supplementary Materials).

β-CA which is by far the best substrate of NIT4 [4] was also transformed by *E. coli* cells containing NitTv1 [18]. However, the cells only transformed β-CA at low rates (<0.2 U mg^−1^ dry cell weight). The purified enzymes were incubated with 25 mM of β-CA and ammonia were determined spectrophotometrically. Thus, the NLase activity of the purified NitTv1 was ca. 94 U mg^−1^ of protein and that of NitAb was ca. 3.5 times lower (Table 2). The difference between the activities of the purified NitTv1 and the whole cells producing this enzyme could be caused by limitations of the substrate transport into the cells. Apparently, the activities of the purified enzymes increased by ca. 40–50% if asparaginase (transforming Asn into Asp and ammonia) was added to the reaction medium. This indicated that the fungal enzymes, similar to plant NIT4 [4], exhibited two activities, which were designated nitrile hydratase (NHase) (β-CA → Asn) and NLase (β-CA → Asp + ammonia) according to [4]. The formation of Asp and Asn from β-CA in absence of asparaginase was observed in TLC using authentic standards (not shown). The NHase:NLase activity ratio was lower in the fungal NLases than in NIT4 from *A. thaliana* or TNIT4A and TNIT4B from *Nicotiana tabacum*. The NLase activity in NitTv1 exhibited a similar *V*_max_ for β-CA as NIT4. In contrast, *V*_max_ of the NLase activity in NitAb was lower than in NIT4. The *V*_max_ values of the NHase activities were lower in both fungal enzymes than in NIT4. The *K*_M_ values were generally higher in the fungal enzymes than in NIT4 (Table 2).

β-CA is a typical substrate of NIT4 but inferior substrate of NIT1-NIT3, while the opposite is true for 3-phenylpropionitrile (PPN). The ratios of activities for β-CA and PPN were lower in the two fungal NLases than in NIT4, but higher than in the NLases from *N. tabacum* (Table 2). The high activity of the fungal NLases for β-CA and the β-CA:PPN activity ratio strongly suggested classifying the fungal NLases as the NIT4-type.

Other nitriles, which were previously identified as substrates of NitTv1, were also tested with the purified enzyme. These were fumaronitrile (FN) and 4-cyanopyridine (4CP). In addition, some representatives of nitriles which were accepted as substrates of plant NLases NIT1/NIT2/NIT3 [9,10] were used. These were allylcyanide (AC), phenylthioacetonitrile (PTAN), 4-phenylbutyronitrile (PBN) and cinnamonitrile (CN). Phenylacetonitrile (PAN) and indole-3-acetonitrile (IAN) as potential precursors of auxins (corresponding carboxylic acids) were also included in this set of substrates. The same substrates were examined with NitAb.

The reactions of 25 mM substrates were run for 5–10 min and the formation of products was examined. The carboxylic acids and amides formed from these substrates were determined by HPLC. The RTs and UV spectra of the products were in accordance with those of authentic standards (not shown). Relative activities were calculated with β-CA as reference substrate (Table 3), for which the total activity (the sum of NLase and NHase activities) was determined by the ammonia assay with asparaginase. The expected molecular masses of the products were confirmed by LC-MS after running the reactions for prolonged times (Table S1 in Supplementary Materials).

Although much lower than for β-CA, the specific activities of NitTv1 for arylaliphatic substrates (CN, PPN, PAN, PTAN) and, especially, FN were significant (ca. 1–11 U mg^−1^ protein). NitAb transformed all these substrates as well, but with lower activities (up to ca. 1U mg^−1^ protein for CN). In contrast, NIT4 only exhibited non-negligible activities for PPN, PAN and methylthioacetonitrile (MTAN) [4] (Table 3).

The temperature and pH dependence of activity and stability of the enzymes was examined with CN (Table 3; Figure S6 in Supplementary Materials). NitTv1 and NitAb produced acid and amide at ca. 0.65 and 2.5 ratio, respectively, from CN. Hence, the assays with CN also enabled to determine the potential effects of temperature and pH on the NHase:NLase activity ratio. This may not be possible with β-CA, as the activity of the asparaginase added in the coupled assay (see above) is also dependent on temperature and pH.

In NitTv1, the temperature optimum was slightly higher for the NHase than NLase activity (30–35 °C vs. 30 °C; Figure S6A in Supplementary Materials). NLase was fairly stable at up to ca. 35 °C but NHase was slightly more stable than NLase (Figure S6B). The pH optimum was ca. 7.5–8.5 for both activities but NHase retained more of its activity at pH 4.5–6.5 than NLase (Figure S6C). Both activities were fairly stable at pH 5.2–9.3 but no activities were found at pH ca. 4 and 11 (Figure S6D). Thus, the reaction conditions (temperature, pH) affected the NHase:NLase activity ratios. Specifically, this effect was significant at elevated temperatures or at slightly acidic pH, where this ratio increased (Figure S6A–C).

NitAb was most active at 25-30 °C and exhibited no significant activity at 40 °C (Figure S6E). Its stability was fair at temperatures of up to 35 °C but low or no activity was kept at 40 or 45 °C (Figure S6F). Its pH optimum was ca. 6–8 (Figure S6G). The enzyme was more sensitive to slightly acidic pH than NitTv1, exhibiting no activity at pH around 4.5. The pH stability was fair at pH 5–7 but lower at pH 7–9 (Figure S6H). The differences between the temperature and pH optima and stabilities of the NHase and NLase activities in NitAb (Figure S6E–H) were either not significant or less significant than in NitTv1 (Figure S6A–D).

The temperature optima of NitTv1 (30–35 °C) and especially NitAb (25–30 °C) were lower than those of NIT4 (40 °C). The reaction times used with NitTv1 and NitAb were shorter than with the plant NLases (60 min in NIT1 [10], 20 min in NIT4 [4], 10 min in NitTv1 and NitAb). The data cannot be directly compared due to different conditions but the conclusion on the lower temperature optimum of the fungal NLases is probably justified. The pH optima of the fungal NLases were similar as that of NIT4 (pH 7–9) [4] and lower than that of NIT1 (pH 9) [10]. Stabilities were determined after 24-h incubation at different temperatures in plant NLase NIT1 and the enzyme was found to be fairly stable at up to 35 °C [10]. These data are hardly to be compared with those for fungal NLases due to different reaction conditions. The fungal NLases were fairly stable at up to 25 °C (NitAb) or 35 °C (NitTv1) after 2-h incubation (Table 3). The storage stability of the enzymes was satisfactory. NitTv1 maintained ≥95% residual activity after 15 days and NitAb ca. 42% residual activity after 18 days at 4 °C.

### 2.3. Assessment of the Enzyme Affinities to Various Substrates Using Homology Modeling and Substrate Docking

**Figure 2:**
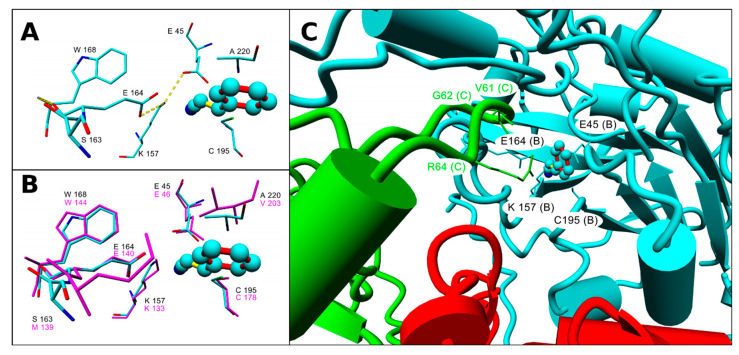
(**A**) Active site residues of NitAb with docked cinnamonitrile (CN): amino acid residues which are within 0.4 nm from ligand or could participate in controlling the nitrilase activity [22] are shown. These residues are also labelled in the multiple sequence alignment (Figure S3 in Supplementary Materials). Hydrogen bonds formed by catalytic residues are shown with yellow dotted lines. (**B**) Overlay of the active site residues of NitAb (element color, black labels) and NitTv1 (magenta color, magenta labels). (**C**) Orientation of monomers in tetrameric NitAb. Active site of monomer B with docked CN is framed by the residues from monomer C (green color) and monomer A (red color). Residues of monomers A, B and C within distance 0.3 nm from docked CN are shown with monomer name in brackets. The catalytic tetrad is shown in monomer B.

The homology model of NitTv1 was constructed previously [18]. Since then, a new template NIT4 in *A. thaliana* (pdb code 6i00) [21] emerged. In this study, this template was used for multiple sequence alignment (Figure S3) along with the previously used template [18], i.e., the NLase from *Synechocystis* sp. (pdb code 3wuy [22]). The new template enabled to refine the modeling of loop HL1 in NitTv1. The homology model of NitAb was constructed analogously. Active sites of NitAb and NitTv1 are similar as in other NLases [18]. The catalytic tetrad in NitAb consists of E45, K157, C195 and E164, and that in NitTv1 of E46, K133, C178 and E140 (Figure 2A,B and Figure S3 in Supplementary Materials).

Similar to NitTv1 [18], NitAb has extended loops HL1, HL2 and HL4. Additionally, it has an extended loop HL1b between helices α3 and α4; this loop was neither found in homologous structures nor in NLases from clade 1, to which NitTv1 belongs. Consensus secondary structure prediction method [23] showed that residues 93–95 (WIE) in this loop have a higher helix propensity.

NLases often form homooligomeric spirals (helices), initiated by ligand binding or C-terminal truncation [3]. However, as this information on the active form of NitAb or NitTv1 has not been available yet, we used both the monomer and an oligomer (tetramer) for the docking studies (Figures S7 and S8 in Supplementary Materials). The recently reported structure of NIT4 [21] allowed a precise modeling of loop HL1 and hence improved prediction and characterization of ligand binding in the NLase oligomers.

Binding Glide standard precision (SP) scores of ligands in the active site of monomers and tetramers of NitTv1 and NitAb are shown in Table 4. All ligands could be docked in the binding pocket of NitAb and NitTv1 (either in monomer or tetramer) with clear preference for arylaliphatic nitriles. In both NitAb and NitTv1 the oligomerization largely improved binding. Docking CN, MTAN, AC and octanenitrile (ON) in NitTv1 and 4CP in NitAb could not be done in monomer, but only in tetramer structure.

**Figure 3:**
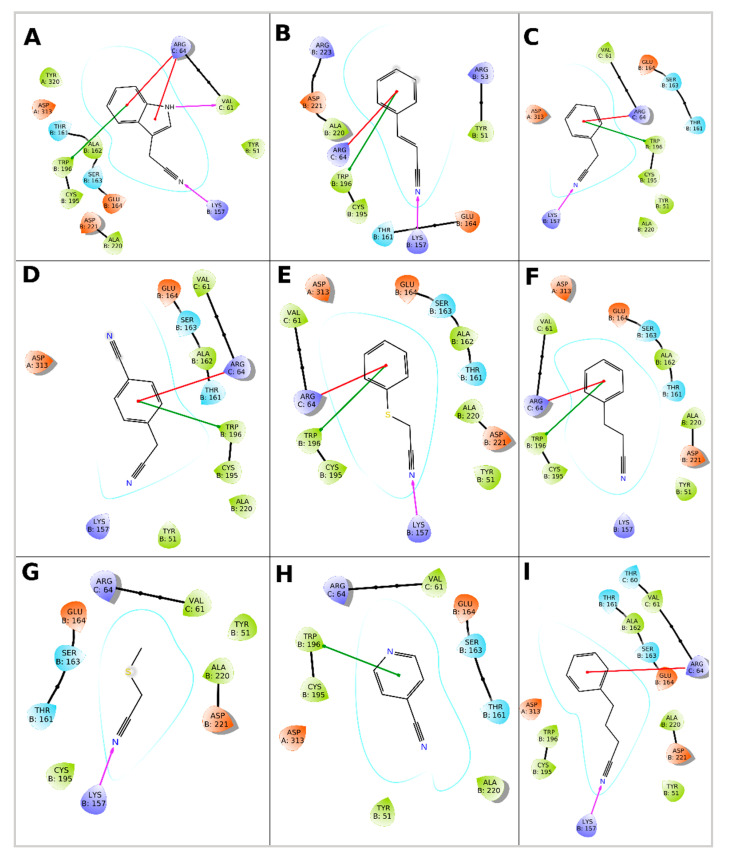
Interaction of NitAb with docked ligands: (**A**)—Indole-3-acetonitrile; (**B**)—cinnamonitrile; (**C**)—phenylacetonitrile; (**D**)—4-cyanophenylacetonitrile; (**E**)—phenylthioacetonitrile; (**F**)—3-phenylpropionitrile; (**G**)—methylthioacetonitrile; (**H**)—4-cyanopyridine; (**I**)—4-phenylbutyronitrile. The monomers are named A, B, C. The ligand is docked in monomer B.

**Figure 4:**
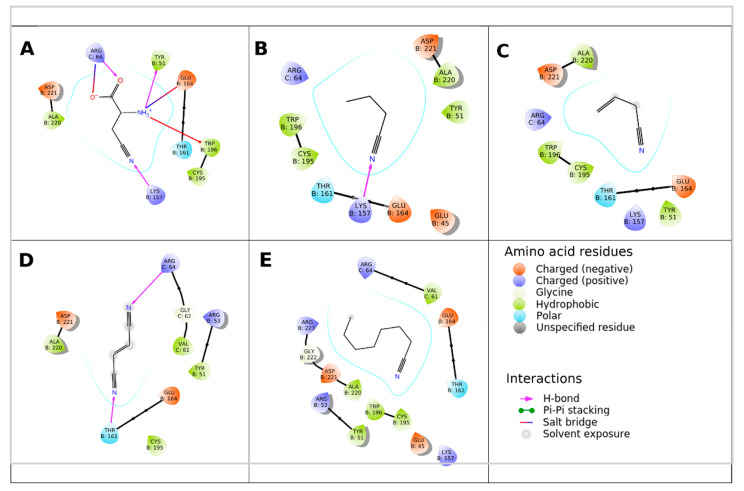
Interaction of NitAb with docked ligands: (**A**)—β-L-cyanoalanine (protonation corresponds to pH 9); (**B**)—butyronitrile; (**C**)—allylcyanide; (**D**)—fumaronitrile; (**E**)—octanenitrile. The monomers are named A, B, C. The ligand is docked in monomer B.

The better binding of arylaliphatic nitriles (Figure 3A–F,I) in comparison with aliphatic nitriles (Figure 3G, Figure 4A–E) in NitAb could be explained by additional interactions formed by the aromatic ring. These were a T-shaped π-stacking with W196 (in the same monomer as used for docking; Figure 3A–F) and a cation—π-stacking with R64 of loop HL1 (in another monomer placed above the active site in the oligomeric structure; Figure 3A–F,I). The former was also found in 4CP (Figure 3H). Binding of 4CP in the NitAb tetramer is influenced by the steric conflict of the pyridine group with R64 (R65 in NitTv1).

Docking of β-CA demonstrated the pH dependence of NitAb affinity for this ligand (Figure 4A). The changes in pH affected the protonation state of the carboxyl and the amino group. This resulted in changing the interactions of this ligand with the active site residues.

The models indicate that the active sites of both NitAb and NitTv1 are big enough to bind bulky substrates. However, we assumed that molecular dynamics (MD) simulation in water will demonstrate more favorable contacts of the smaller substrates such as FN (Figure 4D) or other aliphatic nitriles (Figure 4) with the active site and, hence, their better accommodation in it. We also expected this may explain the differences in the experimentally determined activities of NitTv1 and NitAb towards FN (Section 2.2).

Therefore, we performed short MD simulations of the tetramers. The stability and equilibrium orientation of FN in the active sites of the two enzymes were different (Figure S9 in Supplementary Materials). The binding of FN was more stable with NitTv1 (see root means square deviation (RMSD) in Figure S9), while in NitAb FN left the active site of monomers A and D. Binding of ligand in the active sites of monomers B and C was influenced by interaction with another monomer in NitAb. Hence, it represents the dynamics of the ligand docked in the oligomeric structure, while the ligand dynamics in the monomer is shown for monomers A and D (Figure S9).

From the MD simulations of other representative structures, we could conclude that docking of ligands in NitTv1 is better in the monomer, while the ligand–enzyme complexes formed by NitAb are more stable as tetramers (Figures S10 and S11 in Supplementary Materials).

**Figure 5:**
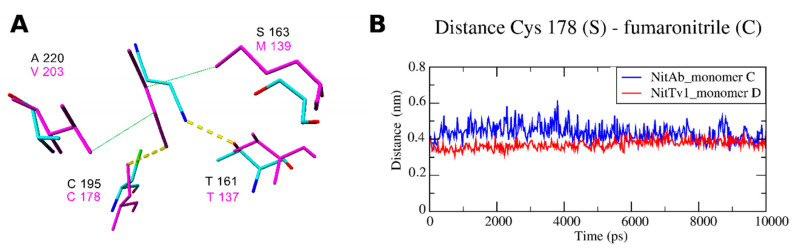
(**A**) Overlay of NitTv1 monomer D (magenta color) and NitAb monomer C (element color) with docked fumaronitrile after 10 ns of molecular dynamics (MD) simulations. The selection of the representative monomers was based on the lowest ligand root means square deviations (RMSD) and distance from catalytic C residue. (**B**) Distance between catalytic C residue and fumaronitrile in representative monomers during MD simulation.

The distance between the S atom of the catalytic C residue and the cyano group of the ligand is an important parameter influencing the enzyme activity [22,24]. FN was shown to be closer to the catalytic C residue in NitTv1 compared to NitAb (Figure 5A,B; Figure S9 in Supplementary Materials). The difference in the equilibrium conformation of FN in these enzymes is determined by some differences in their primary sequences (Figure 5A)—substitution of small A220 in NitAb by the more bulky and hydrophobic V203 in NitTv1, and substitution of polar S163 in NitAb by hydrophobic M139 in NitTv1. The hydrophobic side chains in NitTv1 enable a hydrophobic contact with the double bond in FN (Figure 5). In contrast, FN docked in NitAb often formed a hydrogen bond (HB) with T161 during MD. Namely, this was observed in 65% of the MD simulations (Video V1 in Supplementary Materials). The corresponding T residue is also found in the binding pocket of NitTv1 (T137), but, due to an improved hydrophobic interaction of FN with M139 and V203, HB with FN was present in less than 4% of the MD simulations (Figure 5).

## 3. Discussion

The beginning of plant-NLase studies dates back to the 1960s when the NLase from *Hordeum vulgare* (barley) was described as one of the first NLases ever reported. Similar NLases were then obtained, after some gap in plant NLases studies, from plants belonging to, e.g., *Brassica rapa* (Chinese chabbage) or *Brassica napus* (rapeseed) in the 1980s–1990s. The cloning of NLase genes in the model plant *A. thaliana* and their localization (isoenzymes NIT1–NIT3 on chromosome 3 and isoenzyme NIT4 on chromosome 5) followed by expression of all four isoenzymes in *E. coli* belonged to the key advances in the research of plant NLases [11].

The difference between isoenzymes NIT1–NIT3 mainly resides in the localization of their gene expression. In contrast, their substrate specificities are very similar. They transform arylaliphatic nitriles such as PPN, AC and PTAN at the highest rates [9]; in addition, a high activity for ON was found in NIT1 [10]. β-CA is a poor substrate of NIT1-NIT3 with less than 1% activity relative to PPN [9]. Hence, the physiological roles of NIT1–NIT3 are probably associated with the degradation of nitriles available from glucosinolates (PPN, IAN) and, optionally, their conversion into plant hormones [11,12]. In contrast, NIT4 has activity for β-CA ca. two orders of magnitude higher than NIT1–NIT3 but only very low activities for arylaliphatic nitriles such as PPN or PAN. The specific activities for their preferred substrates—PPN in NIT1 and β-CA in NIT4—were comparable with over 30 U mg^−1^ protein [4,10].

The isoenzymes NIT1–NIT3 are less widespread in plants than the NIT4 isoenzyme, which is in accordance with their substrate specificities and their proposed roles. While glucosinolates, which are degraded to provide some of the arylaliphatic substrates for NIT1–NIT3, are almost only produced in members of order *Brassicales*, among them family *Brassicaceae* (Chinese cabbage, rapeseed) [11], the substrate of NIT4 isoenzymes—β-CA—is widely present in plants as the intermediate of HCN breakdown [12]. Thus, GenBank searches [19] demonstrate that the close homologues of NIT1 almost exclusively originate from order *Brassicales*, while those of NIT4 are widely distributed over flowering plants (*Magnoliophyta*). The NIT1 homologues were largely designated as putative proteins but a number of them were predicted to be NLase 1, 2 or 3 (i.e., NIT1–NIT3 isoenzymes). Many of the NIT4 homologues are designated “bifunctional NLase/NHase NIT4”, “NIT4-like isoform” or similar [19].

Homologues of the plant NLases are also encoded in the genomes of microorganisms (bacteria, fungi). In bacteria, the published sequences with >50% identities (>70% cover) to NIT4 are in the order of thousands [19]. However, few bacterial NLases of this type were studied biochemically.

One of them was from *Pseudomonas fluorescens*, which is a plant symbiont growing on plant surfaces and inside plants [25]. This species contains around one hundred of NIT4 homologues [19]. The activity of the *P. fluorescens* NLase (CAY48939.1) for β-CA was confirmed after expressing the corresponding gene (*pinA*) in *E. coli*. However, the NLase was only examined in crude extracts, as the attempts to purify it in a soluble form were not successful. This NLase enabled *P. fluorescens* to metabolize the toxic β-CA and to utilize it as the source of nitrogen. In addition, *pinA* expression in a NIT4-defficient mutant of *A. thaliana* enabled the plants to cope with exogenous β-CA and improved the root elongation in wild-type plants [25]. The identity of this NLase to the characterized NLase (AAW79573.1) from *P. fluorescens* EBC191 is relatively low (ca. 36%). The latter enzyme is an arylacetoNLase with activity for α-substituted nitriles such as (*R*,*S*)-mandelonitrile [26].

The NIT4 homologue (WP_014332611.1) from *Sinorhizobium fredii*, a nitrogen-assimilating bacterium, was recently purified by us and its activity for β-CA (≈2 U mg^−1^ protein) was determined. Other substrates of this enzyme were e.g., FN, PAN or various arylaliphatic dinitriles [17]. This protein exhibited ca. 58% identity to NIT4. It has over 200 homologues with >50% identities in bacteria such as *Azospirillum*, *Bradyrhizobium*, *Rhizobium* (plant symbionts) or *Achromobacter* (occurring in soil among other wet environments). One of these NLases (WP_011086181.1), from *Bradyrhizobium japonicum* (re-classified as *Bradyrhizobium diazofficiens*), was overproduced and purified. It exhibited its highest specific activity for PPN (ca. 10 U mg^−1^ protein) and also transformed PAN, MTAN and *n*-alkyl nitriles with C_5_–C_7_ chains at significant rates [27]. This enzyme is available from Prozomix (PRO-E0264) [6].

Fungi also contain large numbers of genes coding for homologues of plant NLases but almost nothing was known on the catalytic properties of the corresponding proteins. Only recently, the activity for β-CA was experimentally verified in one of them, NitTv1 [18], using *E. coli* whole cells expressing the *NitTv1* gene. NitTv1 and its homologues form clade 1 in division *Basidiomycota*, subdivision *Agaricomycotina*. The members of this clade (over 80) exhibit 55–88% identities to NitTv1. They were not found in other subdivisions of *Basidiomycota*. These NLases primarily occur in class *Agaricomycetes* but only rarely in other *Agaricomycotina* (class *Dacrymycetes*) [18].

The fungi encoding NitTv1 homologues are largely able to grow on living or dead plants and many of them are plant pathogens. For instance, *Rhizoctonia solani* is a phytopathogenic fungus occurring on agricultural crops; *Dichomitus squalens*, *Trametes* sp. and *Armillaria* sp. are wood-rotting fungi. Several NIT4 homologues were found in each of them. It is possible that phytopathogenic fungi acquired the NIT4-coding genes by horizontal gene transfer and thus gained the ability to detoxify β-CA. NitAb is unique in *Basidiomycota* but it has several hundreds of close homologues in *Ascomycota*. These hypothetical NLases exhibit ca. 50–70% identities to NitAb. They are widely distributed in subdivision *Pezizomycotina*, where they occur in. e.g., the classes of *Dothideomycetes*, *Eurotiomycetes*, *Leotiomycetes* and *Sordariomycetes*.

In this study, we purified and characterized the first fungal NLases with activities for β-CA. Both NitTv1 and NitAb exhibited substrate specificities similar to NIT4, although NitAb is more distant in evolution from NIT4 than NitTv1. However, unlike NIT4, the fungal NLases also transformed some arylaliphatic nitriles known as substrates of NIT1 (PPN, CN, FN, PAN, PTAN) [9,10] at non-negligible rates. PPN and PAN were also transformed by NIT4 [4] but the relative activities of the fungal NLases towards these substrates were slightly higher. However, this must be evaluated with caution due to differing substrate concentrations in assays of different enzymes. The activities for FN or CN, which were relatively high in NitTv1, were not tested with NIT4 [4].

Plant NLases and their fungal analogues exhibit a strong tendency to produce amides. The amide-forming activity was designated NHase [4], although the enzyme is different from the “true” NHases (EC 4.2.1.84). The amide-forming NLases may optionally replace the “true” NHase in biocatalysis. The genuine NHases are metalloenzymes and tend to be unstable. They are also rarely enantioselective. In contrast, NLases are often more stable and many of them are enantioselective. The ratio of NHase:NLase activities depends on enzyme and substrate. Effect of pH and temperature on amide formation was mainly observed in NitTv1. Previously, the NHase:NLase ratio was found to be influenced by temperature and pH in the NLase from *P. fluorescens* [28]. The amide formation was further increased in this NLase by enzyme engineering. The mutants were suitable for the production of (*S*)-mandelamide from (*R*,*S*)-mandelonitrile [29].

The effects of pH and temperature on the enzyme activities and stabilities were also taken into account, while assessing the enzymes’ utility as catalysts. The enzymes largely exhibited moderate thermal stabilities as many NLases from plants and mesophilic bacteria. NLases rarely exhibit higher thermostabilities except for those from extremophiles such as *Pyrococcus abyssi*; however, the disadvantage of this NLase is its narrow substrate specificity [3]. However, the thermal stabilities could be improved by various techniques such as co-expression of GroEL/ES chaperone genes from *Rhodococcus ruber* [30] or by enzyme cross-linking with acryloyl-crosslinked cellulose dialdehyde [31].

The pH optima were similar as in other NLases [3], i.e., near neutral pH. However, NitTv1 exhibited a very good pH resistance. Previously, the examination of a large set of enzymes indicated that pH activity and stability profiles are in correlation. However, this rule may not be valid for unnatural media [32]. Thus, NiTv1 was more stable than active at slightly acidic or slightly alkaline pH. This could be due to the different protocols used in activity and stability assays (preincubation is carried out without cosolvent or substrate in the latter). In addition, enzyme-specific pH effects are plausible in NLases, such as the transformation of short spirals into long rods upon pH change from 8.0 to 5.4 in NLase (cyanide dihydratase) from *Bacillus pumilus* [33].

The Asn:Asp ratios in the conversions of β-CA were lower in NitTv1 or NitAb than NIT4. Significant amounts of amides were also produced from other substrates (FN, CN, PTAN, IAN, 4CP) by the fungal NLases as determined by LC-MS. Previously, the formation of amide (Asn) was only monitored for β-CA in NIT4, while the activities for other substrates were examined using an ammonia assay [4]. Using this assay may lead to underestimating the activities for some substrates, as the potential formation of amide is not taken into account. Amides were prepared from several substrates (fluoronitriles, nitroacrylonitrile, FN) using NLase NIT1; the product of FN was cyano amide [10]. In addition, NIT1 enabled to obtain (*R*)-2-fluoroarylacetamides from the corresponding racemic nitriles [34].

NLases are supposed to have important roles in the detoxication and utilization of nitriles, plant defense, fungal pathogenicity and plant growth promotion [12]. The examination of the biochemical properties of fungal NLases shed light on some possible mechanisms of fungus-plant interactions. These NLases are able to detoxify β-CA and to convert it into utilizable metabolites (Asp, Asn, ammonia) in vitro. The NLase activity in *P. fluorescens* was found to enable this bacterium to grow in the presence of β-CA and to colonize the plants which produce it [25]. Such interactions are also possible between plants and fungi which grow or parasite on them. Analogously, the studied NLases may enable fungi to detoxify and utilize other plant nitriles such as PPN, PAN, PTAN or IAN. Furthermore, they can convert PPN, PAN or IAN into plant hormones (3-phenylpropionic acid, phenylacetic acid, indole-3-acetic acid) supporting plant growth and creating plant surfaces for colonization by fungi. Certainly, the in vitro examination of the purified enzymes will have to be followed by investigation of the NLase in vivo roles in fungi and fungus-plant systems.

## 4. Materials and Methods

### 4.1. Chemicals

Nitriles (substrates) and authentic standards of carboxylic acids and amides (reaction products) were from standard commercial sources except for (2*E*)-3-cyanoacrylamide and (2*E*)-3-cyanoacrylic acid—the products of transformation of FN, which were previously prepared by us [17].

### 4.2. Analysis of Protein Sequences

The GenBank searches were performed with the program BLAST [19]. The phylogenetic tree of NLases in *Agaricomycotina* was constructed using BLAST pairwise alignments in Blast tree view [19]. Multiple alignments were done in COBALT [35].

### 4.3. Homology Models and Ligand Docking

The model of NitTv1 was previously constructed [18] and the model of NitAb was made analogously. The templates for homology modeling of NitAb were the recently released cryo-EM structure of NIT4 from *A. thaliana* (pdb code 6i00 [21]) and crystal structure of the NLase from *Synechocystis* sp. (pdb code 3wuy [22]). The recent 6i00 structure allowed to refine modeling of loop HL1. The previously modeled NitTv1 structure was improved according to its closer homologue 6i00 [21]. Multiple sequence alignment was constructed with T-Coffee server [36] and used for NitAb model construction, and the refinement of the complete models and loops using MODELLER 9.16 [37] (Appendix A). Program Glide-v7.8 included in Schrödinger package [38,39] was used for rigid receptor docking in the active site of monomer B. The docking grid was defined based on the catalytic tetrad and extended by 0.1 nm in each direction. Additional positional constraints were applied during docking: carbon in cyano group of substrate has to be within 0.31 nm from the sulfur atom of catalytic C residue. (A more soft restraint of 0.35 nm was used for FN.) The ligand orientations were ranked by Glide SP scores. For further MD simulations the best scored poses (i.e., those with the lowest scores) were selected. MD simulations of selected ligand-enzyme complexes were run in YASARA [40] in explicit TIP3P water with YASARA2 force field at NPT ensemble (300 K); protonation states of residues were assigned by YASARA at pH 7 [41].

### 4.4. Genes and Strains of Microorganisms

Genes *nitTv1* and *nitAb* were synthesized by GeneArt (ThermoFisher Scientific) according to sequences published in the GenBank and optimized for *E. coli* in terms of preferential codon usage. The expression strain was *E. coli* Origami B (DE3) harboring pET22b(+) expression system. The expression vectors pET22b(+) carrying gene *nitTv1* or *nitAb* were prepared by GeneArt and the competent cells were transformed with these vectors to produce the proteins fused with a *C*-terminal His_6_-tag as described previously [17].

### 4.5. Enzyme Overproduction and Extraction

Overexpression of the genes was induced with 0.02 mM IPTG as described previously [17]. However, the chaperone production was not induced (L-arabinose was not added to the cultivation medium). After overnight cultivation in 2 × YT medium at 20 °C, the cells (ca. 3 mg dry cell weight mL^−1^) were centrifuged, washed with 20 mM Na/Na phosphate buffer, pH 7.8, containing 0.1 mM of phenylmethylsulfonyl fluoride (buffer P), resuspended in the same buffer to ca. 30 mg dry cell weight mL^−1^ and sonicated (9 × 30 s, 4 °C, 20% of the maximum power output) using ultrasonic homogenizer Bandelin Sonopuls HD2200 (Berlin, Germany). Cell debris was removed by centrifugation (27,000× *g*, 4 °C, 20 min) to give cell-free extract (CFE).

### 4.6. Enzyme Purification

Protein purification was carried out as described previously [17] with minor modifications. Typically, ca. 20 mL the CFE was mixed with 6 mL of TALON Resin (≈50% *w*/*v*; Clontech Laboratories Inc., Palo Alto, CA, USA). The protein load was ca. 50 mg of protein mL^−1^ of resin. The following steps were performed at 4 °C. CFEs were incubated with TALON for 2 h. The resin was then distributed into 2 mL Disposable Gravity Columns (TaKaRa Bio Inc., Shiga, Japan). The non-specifically bound protein was washed out with 5 mM imidazole in buffer P supplemented with 300 mM NaCl and the NLase was eluted with 100 mM imidazole in the same buffer. The NLase was concentrated on Amicon^®^ Ultra-30K at 4000× *g* and 4 °C, and buffer P was exchanged for 50 mM Tris buffer, pH 8.0, with 150 mM NaCl (buffer T), to give solutions containing, typically, ca. 10 mg of protein mL^−1^. Protein was determined using Bradford reagent (Bio-Rad, Hercules, CA, USA). SDS-PAGE was run in 10% polyacrylamide gels with 11–245 kDa markers (Color Prestained Protein Standard, Broad Range; New England BioLabs Inc., Ipswich, MA, USA).

### 4.7. Enzyme Assays

The standard assays were performed using 1.5 mL Eppendorf tubes with 0.5 mL of 25 mM substrate (from 500 mM stock solution in 200 mM Tris/HCl buffer, pH 8.0, for 4CP or in methanol for other substrates) in buffer T at 30 °C and shaking (Thermomixer Eppendorf Compact, 850 rpm). The reactions were started after 5-min preincubation by adding the purified enzyme (ca. 0.01–0.10 mg of protein) and quenched after 5–10 min with 0.05 mL of 2 M HCl. After centrifugation, the nitrile, carboxylic acid and amide concentrations in the supernatants were analyzed by HPLC [18]. Ammonia formed from β-CA was determined spectrophotometrically [42]. Optionally, asparaginase from *E. coli* (Sigma-Aldrich; 4 U) was added to the reaction mixtures with β-CA to transform Asn into Asp and ammonia, and ammonia was determined in the same way. β-CA, Asp and Asn were detected by TLC [43]. For LC-MS analysis (see below), the reactions were run analogously but for 2 h.

The relative activities were determined from the concentrations of ammonia (for β-CA; in presence of asparaginase) or carboxylic acids/amides (for other nitriles) produced from 25 mM substrate under standard conditions. To determine kinetic data, the reactions were run analogously but with 1–25 mM of β-CA. *V*_max_ and *K*_M_ were calculated using program MyCurveFit [44].

The temperature and pH optima were determined with 25 mM CN. The standard assays were modified as follows: To determine the temperature optima, the assays were performed at 20–50 °C in buffer T (pH 8.0). To determine the pH optima, the assays were performed at 30 °C and pH 4.0–11.2 in Britton–Robinson buffers consisting of solution A (phosphoric acid, acetic acid, boric acid; 40 mM each) and B (0.2 M NaOH) at different ratios. To determine the temperature stabilities, the enzymes were incubated at 20–50 °C in buffer T (8.0) at shaking (850 rpm) for 2 h; then the reaction mixtures were pre-incubated at 30 °C for 10 min and the assay was performed under standard conditions. To determine the pH stabilities, the enzymes were incubated at pH 4.0–11.2 in Britton–Robinson buffers (see above) at 30 °C at shaking (850 rpm) for 2 h; then a 3 fold volume of 250 mM Tris/HCl buffer, pH 8.0, was added, and the assay was performed under standard conditions. The activities were calculated from the concentration of cinnamic acid (NLase activity) and cinnamamide (NHase activity).

To determine the storage stability of the NLases, the purified enzymes were kept at 4 °C (on ice) for up to 18 days and their activity was periodically determined with CN at pH 8.0 and 30 °C as described above.

### 4.8. LC-MS Analysis

The LC-MS system (Shimadzu Handels Gmbh, Korneuburg, Austria) was as described previously [18]. The analyses proceeded on a Chromolith RP-C18 (100 × 3 mm) column (Merck KGaA, Darmstadt, Germany) at a flow rate of 0.6 mL min^−1^ in isocratic mode (mobile phase: acetonitrile/water, 20/80, 0.1% formic acid). The MS detector parameters were spray, capillary and tube lens voltages and capillary temperature of 4.5 kV and −3.5 kV, −16 V, −120 V and 250 °C, respectively. Quantitation and molecular mass determination proceeded using the program Labsolutions Version 5.75 SP2 (Shimadzu, Kyoto, Japan).

## 5. Conclusions

A number of plant NLases were previously overproduced and characterized but their homologues in other organisms were largely underexplored except for a few bacterial NLases. The bacterial or fungal homologues are likely to take part in plant–microbial interactions (symbiosis, pathogenesis). In this work, using in vitro studies of the overproduced fungal NLases we elucidated their substrate specificities, which enabled us to hypothesize on their physiological roles. Recently, we were the first to report on the plant NLase homologues in fungi and the activity of one of them for the plant nitrile β-CA. Newly we purified this NLase and one of its fungal homologues, and confirmed their activities for β-CA. It is plausible that these NLases enable fungi to grow on or inside plants despite potentially toxic levels of β-CA in the plant tissues.

In addition, the fungal NLases were found to act on nitriles which are the metabolites of glucosinolates and/or which may serve as precursors of plant hormones. This will provide the fungi with further benefits, such as resistance to other plant nitriles or enlargement of plant surface to colonize. Thus, although similar to NIT4 NLases, the fungal NLases were found to merge, to some extent, the substrate specificities of the plant NIT1–NIT3 and NIT4 types.

The new NLases may be synthetically useful due to their broad range of substrates, their ability to produce amides in addition to carboxylic acids or, mainly in NitTv1, activity and stability over a broad pH range, as well as an excellent storage stability. Their moderate thermal stabilities, however, may have to be improved by, e.g., immobilization. In a good accordance with the in vitro assays, the substrate docking in homology models indicated broad substrate specificities of the enzymes and explained the interactions with substrates in their active sites. These models will be used to predict further substrates of the new NLases.

## Figures and Tables

**Table 1:** Amino acid sequence identities (%) of plant nitrilases NIT1–NIT4 from *Arabidopsis thaliana* and their fungal homologues NitTv1 from *Trametes versicolor* and NitAb from *Agaricus bisporus* var. *bisporus* (sequence coverage (%) in brackets).

	NIT1	NIT2	NIT3	NIT4	NitTv1	NitAb
**NIT1**	-	90.00 (98)	83.82 (100)	68.42 (93)	47.35 (89)	40.36 (86)
**NIT2**		-	83.82 (100)	70.15 (95)	49.37 (90)	39.70 (88)
**NIT3**			-	68.92 (93)	48.44 (88)	39.58 (86)
**NIT4**				-	52.20 (86)	39.88 (84)
**NitTv1**					-	41.96 (96)
**NitAb**						-

**Table 2:** Comparison of the catalytic properties of fungal nitrilases NitTv1 and NitAb and plant nitrilases from *Arabidopsis thaliana* (NIT4) and *Nicotiana tabacum* (TNIT4A, TNIT4B).

Enzyme ^1^	Substrate β-CA					β-CA:PPN Activity	Reference
	Specific Activity [U mg^−1^ protein]		*V*_max_ [U mg^−1^ rotein)] *K*_M_ (mM)		NHase:NLase Activity		
	Asparaginase						
	+ (Total Activity)	− (NLase Activity)	NLase Activity ^2^	NHase Activity ^2^			
**NitTv1**	131.5 ± 0.5 ^3^	94.2 ± 1.0 ^3^	129.8 ± 11.4 7.72 ± 1.82	53.2 ± 9.2 6.74 ± 3.35	0.40 ± 0.02 ^3^	91 ± 3 ^3^	This work
**NitAb**	40.1 ± 0.1 ^3^	26.8 ± 0.4 ^3^	34.8 ± 2.0 7.38 ± 1.40	19.6 ± 2.5 4.97 ± 2.12	0.50 ± 0.02 ^3^	56 ± 4.0 ^3^	This work
**NIT4**	n.d.	31.8 ^4^	110.4 ± 9.6 0.74 ± 0.25	153.0 ± 25.8 0.70 ± 0.25	1.36 ± 0.21 ^4^	119 ± 18 ^4^	[4]
**TNIT4A**	n.d.	n.d.	n.d.	n.d.	0.87 ± 0.04 ^4^	28 ± 8 ^4^	[4]
**TNIT4B**	n.d.	n.d.	n.d.	n.d.	1.06 ± 0.12 ^4^	20 ± 4 ^4^	[4]

^1^*C*-terminal His_6_-tag. ^2^ Asp- and Asn-forming activity was designated NLase and NHase activity, respectively, according to [4]. ^3^ The specific activities were determined with 25 mM of β-CA at pH 8 and 30 °C. ^4^ The specific activities were determined with 3 mM of β-CA at pH 8 and 30 °C. n.d. = no data; β-CA = β-cyano-L-alanine; PPN = 3-phenylpropionitrile; NHase = nitrile hydratase; NLase = nitrilase.

**Table 3:** Comparison of biochemical properties of fungal nitrilases NitTv1 and NitAb and plant nitrilases from *Arabidopsis thaliana* (NIT1–NIT4).

Enzyme	Relative Activity [%]	Optima; Stabilities	Reference
NitTv1 (purified) ^1^	β-CA (100), FN (8.6), 4CP (1.7), CN (1.6), PPN (1.1), PAN (1.0), PTAN (<1), IAN (<1) ^3^	pH 7.5–8.5/30–35 °C; pH 5.2–9.3/≤35 °C	This work
NitAb (purified) ^1^	β-CA (100), CN (3.3), PPN (1.8), PAN (1.1), FN (<1), PTAN (<1), 4CP (<1), IAN (<1) ^3^	pH 6–8/25–30 °C; pH 5–7/≤25 °C	This work
NIT1 (cell extract) ^1^	PPN (100), AC (94), PTAN (68), PAN (5), 4CP (<1), β-CA (<1) ^4^	n.d.; n.d.	[9]
NIT1 (purified) ^2^	PPN (100), ON (40), PBEN (26), PBN (21), BN (14), CN (6.5) ^5^	pH 9, 35 °C; <35 °C	[10]
NIT2 (cell extract) ^1^	PPN (100), AC (100), PTAN (80), PAN (13), 4CP (<1), β-CA (<1) ^4^	n.d.; n.d.	[9]
NIT3 (cell extract) ^1^	PPN (100), PTAN (63), AC (43), PAN (2), 4CP (<1), β-CA (<1) ^4^	n.d.; n.d.	[9]
NIT4 (purified) ^1^	β-CA (100), PPN (0.75), PAN (0.23), MTAN (0.22) ^6^	pH 7–9/40 °C; n.d.	[4]

^1^*C*-terminal His_6_-tag. ^2^*N*-terminal His_6_-tag. ^3^ Determined with 25 mM of substrate at pH 8 and 30 °C. Specific activities of 131.5 and 40.1 U mg^−1^ protein for β-CA (Table 1) were taken as 100% in purified NitTv1 and NitAb, respectively. No significant activities were found with AC, or PBN. ^4^ Determined with 10 mM of substrate at pH 8 and 30 °C. Specific activities of 0.194, 0.198 and 0.192 U mg^−1^ protein for PPN were taken as 100% in NIT1, NIT2 and NIT3 (cell extracts), respectively. The relative activities for selected substrates (approximate values, means of at least three experiments) were retrieved from reference 9. ^5^ Determined with 1.25 mM of substrates at pH 8 and room temperature. Specific activity of 10.2 U mg^−1^ protein for PPN was taken as 100% in purified NIT1. ^6^ The specific activities were determined with 3 mM of substrate at pH 8 and 30 °C. The activity of 31.8 U mg^−1^ protein for β-CA was taken as 100%. n.d. = no data. AC = allylcyanide; BN = butyronitrile; β-CA = β-cyano-L-alanine; CN = cinnamonitrile; FN = fumaronitrile; MTAN = methylthioacetonitrile; ON = octanenitrile; PAN = phenylacetonitrile; PBEN = phenylbut-3-enenitrile; PBN = 4-phenylbutyronitrile; PPN = 3-phenylpropionitrile; PTAN = phenylthioacetonitrile; 4CP = 4-cyanopyridine; NHase = nitrile hydratase; NLase = nitrilase.

**Table 4:** Calculated binding Glide SP scores.

Ligand	Glide SP Score [kcal/mol]			
	NitTv1		NitAb	
	Monomer	Tetramer	Monomer	Tetramer
Cinnamonitrile	n.i. ^1^	−2.569	−2.409	−2.569
Fumaronitrile	−0.150	−0.085	−1.118 ^2^	−0.870 ^2^
Phenylacetonitrile	−3.838	−4.639	−4.170	−4.669
3-Phenylpropionitrile	−3.490	−3.571	−3.146	−4.099
4-Cyanopyridine	−3.260	n.i. ^1^	n.i. ^1^	−3.057
Phenylthioacetonitrile	−3.591	−3.602	−3.453	−4.343
Methylthioacetonitrile	n.i. ^1^	−3.897	−3.694	−3.250
β-Cyano-L-alanine	−2.713	−2.758	−2.416	−2.557
Indole-3-acetonitrile	−4.310	−5.798	−4.112	−5.718
4-Phenylbutyronitrile	−4.201	−3.873	−3.072	−2.932
4-Cyanophenylacetonitrile	−3.144	−4.528	−3.467	−4.389
Allylcyanide	n.i. ^1^	−1.004	−1.487	−1.355
Butyronitrile	−2.978	−3.477	−3.299	−3.538
Octanenitrile	n.i. ^1^	−1.073	−0.651	−0.312

^1^ n.i. = not identified (no position with a negative binding score was found for the ligand). ^2^ Docking of ligand was only possible with increased distance (0.35 nm) between the catalytic C residue (sulfur atom) and the ligand (cyano group). SP = standard precision.

**Figure A1:**
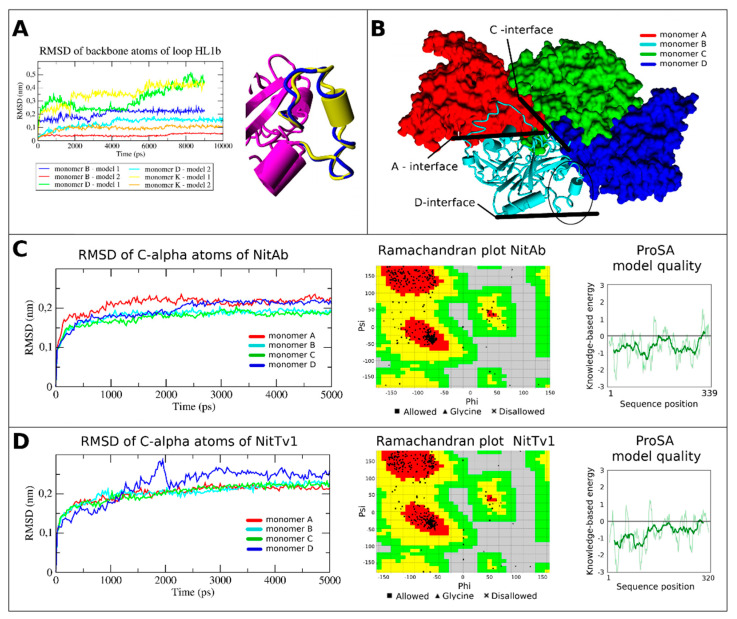
(**A**) Refinement of loop HL1b in NitAb. (Left) Root means square deviations (RMSD) of backbone atoms of loop HL1b (residues 82–110) during loop HL1b equilibration. (Right) Structural alignment of loop HL1b from model2 (monomer B) before refinement (blue) and after it (yellow); the rest of monomer B is colored magenta (right). (**B**) Model of NitAb tetramer. Monomers A (red), C (green) and D (blue) are represented by molecular surface, monomer B (cyan) by secondary structure in cartoon style. Interfaces between the monomers are marked with black lines; position of loop HL1b is marked with a black ellipse. (**C**,**D**) Analysis of model quality and refinement of NitAb (panel C) and NitTv1 (panel D) models. (Left) RMSD of C-alpha atoms of monomers during the complete model refinement. (Center) Ramachandran plot calculated for the most stable monomer B after refinement. (Right) The red, yellow, green and grey regions represent the favored, allowed, generously allowed and disallowed values, respectively, of the backbone phi-psi dihedral angles. Residues are marked in black. Smoothed ProSa-web plot of residue energies. Light green: energies averaged over 10 residues, dark green: energies averaged over 40 residues).

## Supplementary Materials

The following are available online, Figure S1: Phylogenetic tree of plant nitrilase homologues in *Basidiomycota*, Figure S2: Nitrilase from *Agaricus bisporus* (NitAb): Gene sequence (optimized) and protein sequence, Figure S3: Multiple sequence alignment used for homology modeling, Figure S4: Scheme of the enzyme preparation protocol, Figure S5: MALDI analysis of purified nitrilases NitTv1 and NitAb, Figure S6: Effect of temperature and pH on activity and stability of nitrilases NitTv1 and NitAb, Figure S7: Proposed tetrameric structure of NitAb, Figure S8: Models of NitAb, Figure S9: Plots of root means square deviations (RMSD) and distance between sulfur atom of catalytic C residue and cyano group carbon of fumaronitrile during molecular dynamics simulation of nitrilases NitAb and NitTv1, Figure S10: Root means square deviations (RMSD) and distance between catalytic C (sulfur atom) and ligand (cyano group carbon) of selected (A) aromatic and (B) aliphatic ligands during molecular dynamics simulation, Figure S11: Comparison of representative distances between catalytic C (sulfur atom) and ligand (cyano group carbon) of selected aromatic (A) and aliphatic (B) ligands during molecular dynamics simulation, Table S1: LC-MS analysis of the reaction mixtures from transformations of 3-phenylpropionitrile (PPN), cinnamonitrile (CN), phenylthioacetonitrile (PTAN) and 3-indoleacetonitrile (IAN), Video V1: Molecular dynamics simulation of fumaronitrile in NitAb active site.

## Appendix A

### Homology Modeling and Loop Modeling

**A1. Modeling of loop HL1b in NitAb.** Loop HL1b is a 25 amino acid long loop between helices α3 and α4 in NitAb (Figure S3). It was not found in any homologous structure. Due to the length of HL1b, the initial model of NitAb was constructed withoutit; later this loop was added during loop modeling. Secondary structure prediction revealed that sequence R^91^LWIEK^96^ formed a secondary structure. The majority of the methods used (Jpred 24], DSC [45], PHD [46]) indicated a higher probability of its helical structure. The combined MLRC approach [47] predicted ^92^WIE^95^ to form a beta-sheet in this region with the highest probability. Different algorithms were used for modeling of loop HL1b—ab initio approach using MODLOOP [48] and the knowledge-based approach implemented in 1 [49,50]. Two best scored loop HL1b models built by these programs demonstrated a helical-like structure of this loop.

NLases usually form higher oligomers [3]. The oligomerization state of NitAb was proposed based on the best template—6i00 from *Arabidopsis thaliana* [21], and constructed by multiple structure alignment implemented in YASARA [40,51].

The proposed tetramer and dodecamer are shown in Figures S7 and S8A,B, respectively. Loop HL1b belongs to D-interface (nomenclature according to [21]). Hence formation of NLase dimeric/tetrameric structure is not restricted by loop conformation, whereas longer helical structures (with more than 10 monomers) sterically restrain the allowed conformational space for HL1b loop. As a minimum model required to effectively restrain the loop, a trimer was selected which was formed by B, D and K monomers (Figure S8C).

Poor contacts between atoms of the amino acid residues in the models were resolved in YASARA. Loop HL1b was optimized and the best model selected. Energy minimization and equlibration were made with YASARA by standard protocol in TIP3P water, YASARA2 force field [40] with periodic boundary conditions followed by short MD simulation (10 ns). During MD simulation, loop HL1b and solvent were allowed to move, while the rest of the system was fixed. RMSDs of backbone atoms in different models of the loop (models 1 and 2 made by different methods) during loop optimization are shown in Figure A1A. RMSD of backbone atoms in loop HL1b in model 2 is below 0.3 nm and it is already stable after 4 ns. Hence, monomer B of model 2 after 5 ns of MD simulation was selected for a further refinement of the NitAb model (Figure A1).

**A2. Refinement of NitTv1 and NitAb models.** The refined models of NitTv1 and NitAb were tetramers consisting of monomers A, B, C and D (Figures S7 and S8 in Supplementary Material). This configuration reflects the fact that the entrance to the active site is close to the C-interface (Figure A1B).

Model refinement was accomplished in YASARA (see Figure A1). Within the complete model refinement, the active site residues (labeled in multiple sequence alignment in Figure S3) were free during the energy minimization but fixed during MD simulations. The time required for the refinement was determined from backbone RMSD calculated in YASARA (Figure A1C,D). The modeled complex was already equilibrated after 3 ns of MD simulation. Monomers B and C were the most stable according to RMSD calculations. Monomer B was selected for further docking and quality assignment.

The quality of the refined models was evaluated using VADAR [52] and ProSA [53]. For more than 88% and 87% residues in the models of NitAb and NitTv1, respectively, the backbone dihedral angles were in favorable regions in the Ramachandran plot (Figure A1C,D). Z-Scores were −7.66 and −8.08 for the NitTv1 and NitAb model, respectively. Both values were within the range allowed for crystal structures.
