# Supplementary material for: Plant Nitrilase Homologues in Fungi: Phylogenetic and Functional Analysis with Focus on Nitrilases in Trametes versicolor and Agaricus bisporus
Source: Molecules. 2020 Aug 25;25(17):3861. doi: 10.3390/molecules25173861 (PMC7503981; doi:10.3390/molecules25173861)
Supplement: Supplementary file 1 [file molecules-25-03861-s001.pdf]

Supplementary Materials

Plant Nitrilase Homologues in Fungi: Phylogenetic and Functional Analysis with Focus on Nitrilases in *Trametes versicolor* and *Agaricus bisporus*

Lenka Rucká [1], Natalia Kulik [2], Petr Novotný [3], Anastasia Sedova [3,4], Lucie Petrásková [3], Romana Příhodová [3], Barbora Křístková [3], Petr Halada [5], Miroslav Pátek [1], Ludmila Martínková [3*]

[1] Laboratory of Modulation of Gene Expression, Institute of Microbiology, Czech Academy of Sciences, Vídeňská 1083, CZ-142 20 Prague, Czech Republic; rucka@biomed.cas.cz (L.R.); patek@biomed.cas.cz (M.P.)

[2] Centre for Nanobiology and Structural Biology, Institute of Microbiology, Czech Academy of Sciences, Zámek 136, CZ-373 33 Nové Hrady, Czech Republic; kulik@nh.cas.cz

[3] Laboratory of Biotransformation, Institute of Microbiology, Czech Academy of Sciences, Vídeňská 1083, CZ-142 20 Prague, Czech Republic; petr.novotny@biomed.cas.cz (P.N.); anastasia.sedova@biomed.cas.cz (A.S.); petraskova@biomed.cas.cz (L.P.); romana.prihodova@biomed.cas.cz (R.P.); barbora.kristkova@biomed.cas.cz (B.K.)

[4] Faculty of Biomedical Engineering, Czech Technical University in Prague, nám. Sítná 3105, CZ-272 01 Kladno, Czech Republic; anastasia.sedova@biomed.cas.cz (A.S.)

[5] Laboratory of Structural Biology and Cell Signaling, BioCeV-Institute of Microbiology, Czech Academy of Sciences, Průmyslová 595, CZ-252 50 Vestec, Czech Republic; halada@biomed.cas.cz (P.H.)

* Correspondence: martinko@biomed.cas.cz (L.M.)

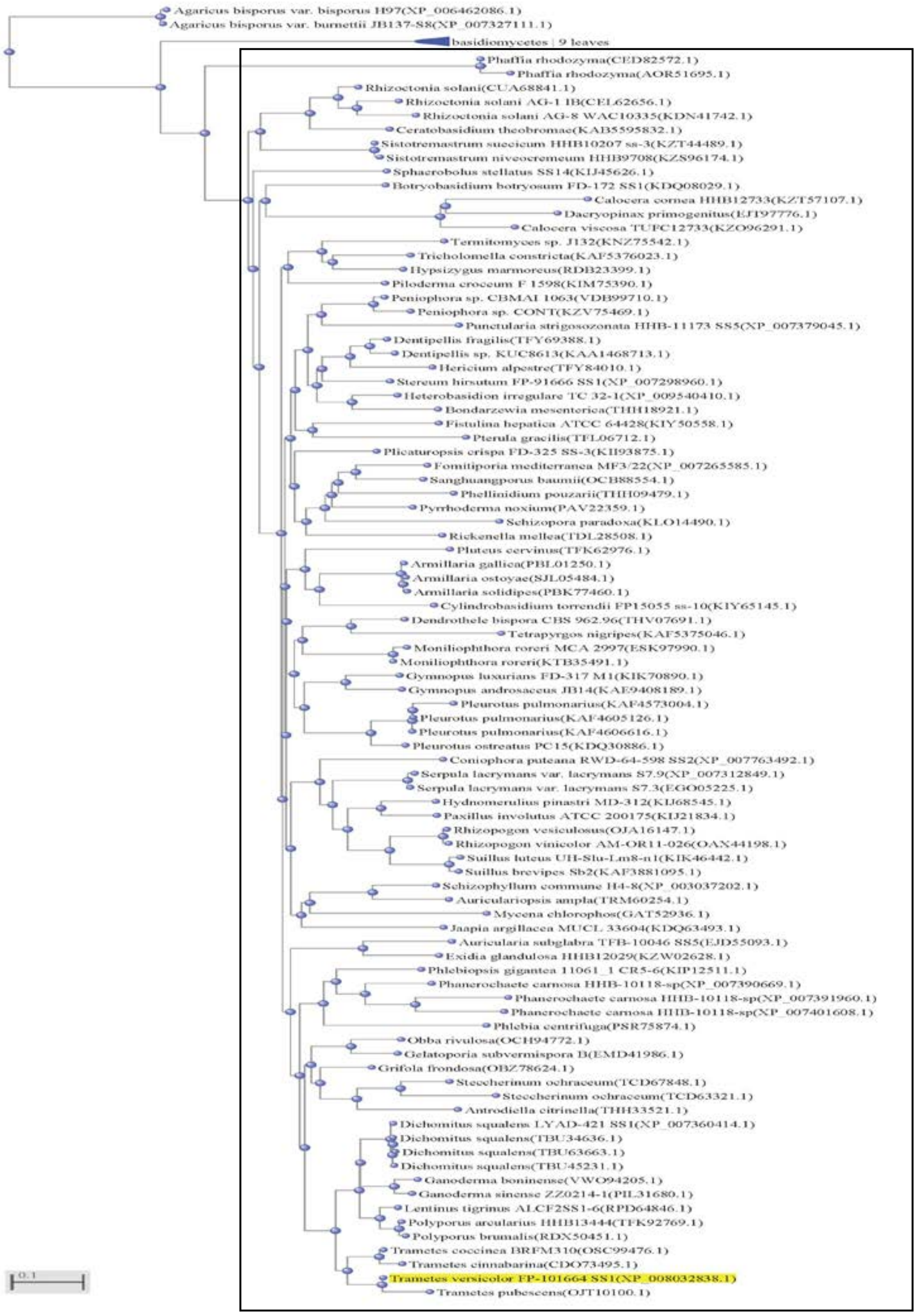

Figure S1. Phylogenetic tree of plant nitrilase homologues in *Basidiomycota* (clade 1 [18] in frame; nitrilase NitTv1 (template) highlighted). See main text for reference.

```
      M P Q T L R V A V S Q S H T L S T T S A T L
1.    CATATGCCGCAGACACTGCGTGTTGCAGTTAGCCAGAGTCATACCCTGAGCACCACCAGTGCAACCCTG
      S A L E Q T V K S A K L Q N D I D L I L F P E
70.   AGCGCACTGGAACAGACCGTTAAAAGCGCAAAACTGCAGAATGATATTGATCTGATTCTGTTCCCGGAA
      A Y L G G Y P R A A S F G A T V G S R S P Q G
139.  GCCTATTTAGGTGGTTATCCGCGTGCAGCAAGCTTTGGTGCAACCGTTGGTAGCCGTAGTCCGCAGGGT
      R E Q F L H Y F K D A V D L G D T P Q G A G R
208.  CGTGAACAGTTTCTGCATTATTTCAAAGATGCAGTGGATCTGGGTGATACACCGCAAGGTGCAGGTCGT
      L W I E K R L E M P S S G D V R G D G T R E V
277.  CTGTGGATTGAAAAACGTCTGGAAATGCCGAGCAGCGGTGATGTTCGTGGTGATGGCACCCGTGAAGTT
      L E R I A K E T G V F V V T G L M E R S G G T
346.  CTGGAACGTATTGCAAAAGAAACCGGTGTTTTTGTTGTTACCGGTCTGATGGAACGTAGCGGTGGCACC
      L Y C A V V Y V C P R L G I V G K R R K V M P
415.  CTGTATTGTGCAGTTGTTTATGTTTGTCCGCGTCTGGGTATTGTTGGTAAACGTCGTAAAGTTATGCCG
      T A S E R L I W G Q G Q P S S L R A I T T T I
484.  ACCGCAAGCGAACGTCTGATTTGGGGTCAGGGTCAGCCGAGCAGTCTGCGTGCAATTACCACCACCATT
      K G V Q I T L A A A I C W E N Y M P L L R Q S
553.  AAAGGTGTTCAGATTACCCTGGCAGCAGCAATTTGTTGGGAAAACTATATGCCGCTGCTGCGTCAGAGC
      L Y S Q N V N L Y L A P T A D G R D T W L S L
622.  CTGTATAGCCAGAATGTTAATCTGTATCTGGCACCGACCGCAGATGGTCGTGATACCTGGCTGAGCCTG
      M Q T V A I E G R C I V L S A N Q C Y T K D D
691.  ATGCAGACCGTTGCAATTGAAGGTCGTTGTATTGTTCTGAGCGCAAATCAGTGTTATACCAAAGATGAT
      L P E W I T Q Q E K D A F D A D E P I S R G G
760.  CTGCCGGAATGGATTACCCAGCAAGAAAAAGATGCATTTGATGCCGATGAACCGATTAGCCGTGGTGGT
      S C I I T P M G K V L A G P L W N E K G G L L
829.  AGCTGTATTATTACCCCGATGGGTAAAGTTCTGGCAGGTCCGCTGTGGAACGAAAAAGGTGGTCTGCTG
      F A D V D F D D C I R G R L D L D V A G S Y S
898.  TTTGCAGATGTGGATTTTGATGATTGTATTCGTGGTCGTCTGGATCTGGATGTTGCAGGTAGCTATAGC
      R N D A F K L T V E G L D L S P P V
967.  CGTAATGATGCATTTAAACTGACCGTTGAAGGCCTGGATCTGAGCCCTCCGGTTCTCGAG
```

Figure S2. Nitrilase from *Agaricus bisporus* (NitAb): Gene sequence (optimized) and protein sequence.

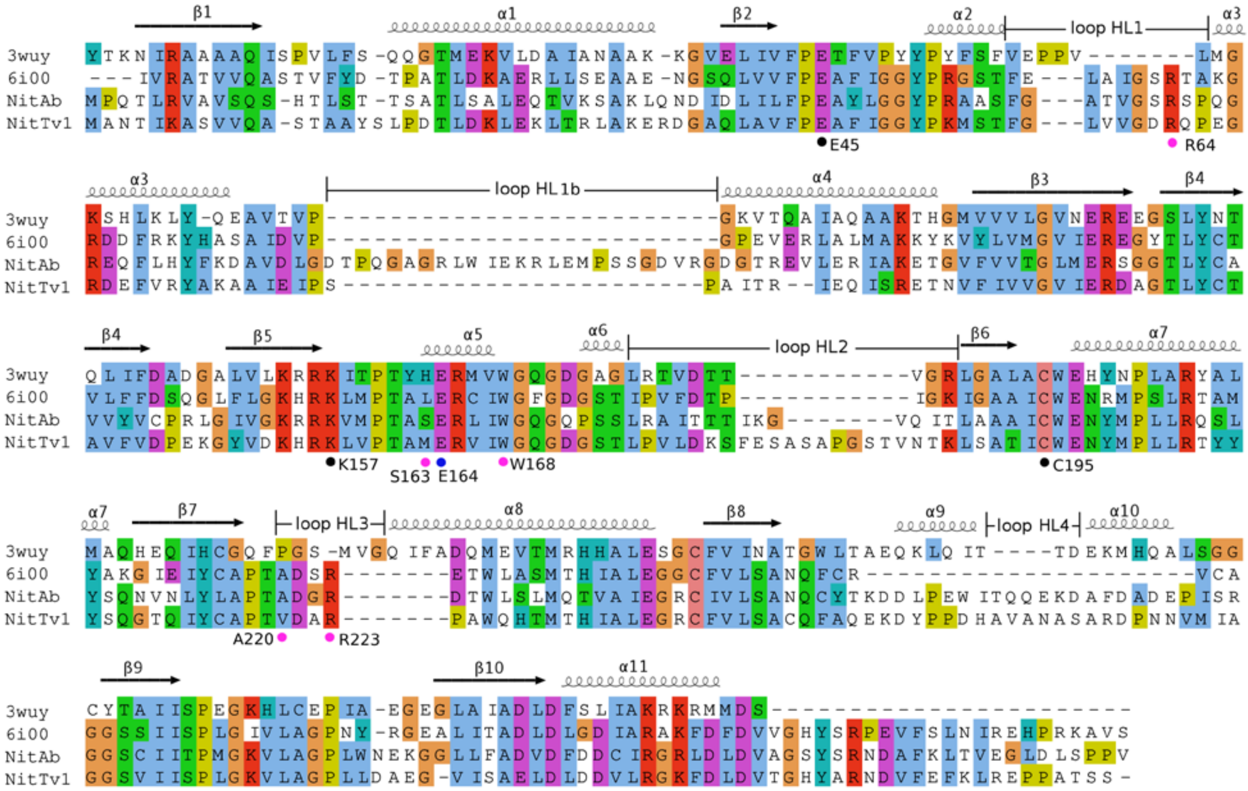

Figure S3. Multiple sequence alignment used for homology modeling. Secondary structure elements are assigned according to 3wuy [23]. The catalytic triad (E, K, C) is labeled with black dots, an additional important E residue is marked with a blue dot. Amino acid residues proposed to be important for substrate recognition are marked with cyan dots. The numbers of residues below the alignment correspond to NitAb. Loops are labeled HL1-HL4 in agreement with [18]. See main text for reference.

Nitrilase overproduction

Escherichia coli Origami B (DE3)
pET22b(+) – *nit* genes clone into *NdeI* and *XhoI* sites
200 mL 2xYT medium + 0.02 mM IPTG, 20 °C, 20 h

↓ Whole cells

Protein extraction

Cells in 20 mM Na/Na phosphate buffer, pH 7.8, 0.1 mM of phenylmethylsulfonyl fluoride (buffer P)
Sonication (9 x 30 s, 4 °C)
Centrifugation (27,000 g, 4 °C, 20 min)

↓ Cell-free extract

Nitrilase purification

Cobalt affinity chromatography (Talon®)
Binding (buffer P, 4°C, 2 h)
Elution of non-specifically bound protein (buffer P + 300 mM NaCl + 5 mM imidazole, 4 °C)
Elution of nitrilase (buffer P + 300 mM NaCl + 100 mM imidazole, 4 °C)
Concentration (Amicon® Ultra-30K, 4,000 g, 4 °C)
Buffer exchange for 50 mM Tris/HCl, pH 8.0, 150 mM NaCl

↓ Purified enzyme

Nitrilase activity assay

$RCOOH + NH_3 \leftarrow RCN \rightarrow RCONH_2$

RCOOH (HPLC) + NH_3 (spectrophotometry) ← RCN → $RCONH_2$ (HPLC)

Figure S4. Scheme of the enzyme preparation protocol.

NitTv1

MANTIKASVVQASTAAYSLPDTLDKLEKLTRLAKERDGAQLAVFPEAFIGGYPKMSTFGLVVGDRQPEGR
DEFVRYAKAAIEIPSPAITRIEQISRETNVFIVVGVIERDAGTLYCTAVFVDPEKGYVDKHRKLVPTAME
RVIWGQGDGSTLPVLDKSFESASAPGSTVNTKLSATICWENYMPLLRTYYYSQGTQIYCAPTVDARPAWQ
HTMTHIALEGRCFVLSACQFAQEKDYPPDHAVANASARDPNNVMIAGGSVIISPLGKVLAGPLLDAEGVI
SAELDLDDVLRGKFDLDVTGHYARNDVFEFKLREPPATSS

NitAb

MPQTLRVAVSQSHTLSTTSATLSALEQTVKSAKLQNDIDLILFPEAYLGGYPRAASFGATVGSRSPQGRE
QFLHYFKDAVDLGDTPQGAGRLWIEKRLEMPSSGDVRGDGTREVLERIAKETGVFVVTGLMERSGGTLYC
AVVYVCPRLGIVGKRRKVMPTASERLIWGQGQPSSLRAITTTIKGVQITLAAAICWENYMPLLRQSLYSQ
NVNLYLAPTADGRDTWLSLMQTVAIEGRCIVLSANQCYTKDDLPEWITQQEKDAFDADEPISRGGSCIIT
PMGKVLAGPLWNEKGGLLFADVDFDDCIRGRLDLDVAGSYSRNDAFKLTVEGLDLSPPV

Figure S5. MALDI analysis of purified nitrilases NitTv1 and NitAb. The peptides found in NitTv1 (21; sequence coverage 71%) and NitAb (23; sequence coverage 63%) are underlined.

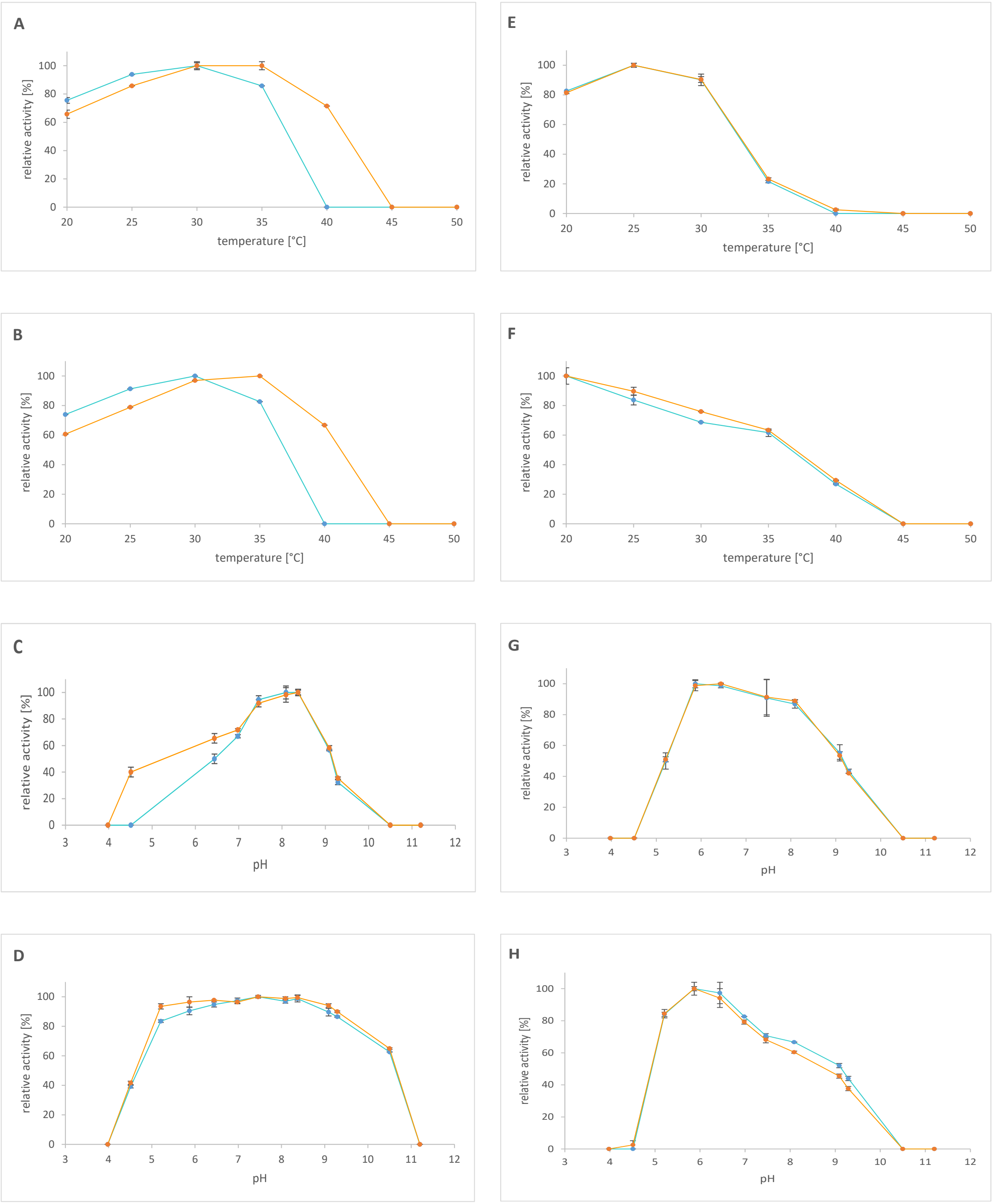

Figure S6. Effect of temperature and pH on activity and stability of nitrilases NitTv1 (left) and NitAb (right). Relative activities (A,E) and stabilities (B, F) at different temperatures. Relative activities (C, G) and stabilities (D, H) at different pH. The activities were determined with cinnamonitrile. The acid-forming activity (nitrilase) and amide-forming activity (nitrile hydratase) are marked in blue and orange, respectively. See main text (section 4.7.) for experimental details.

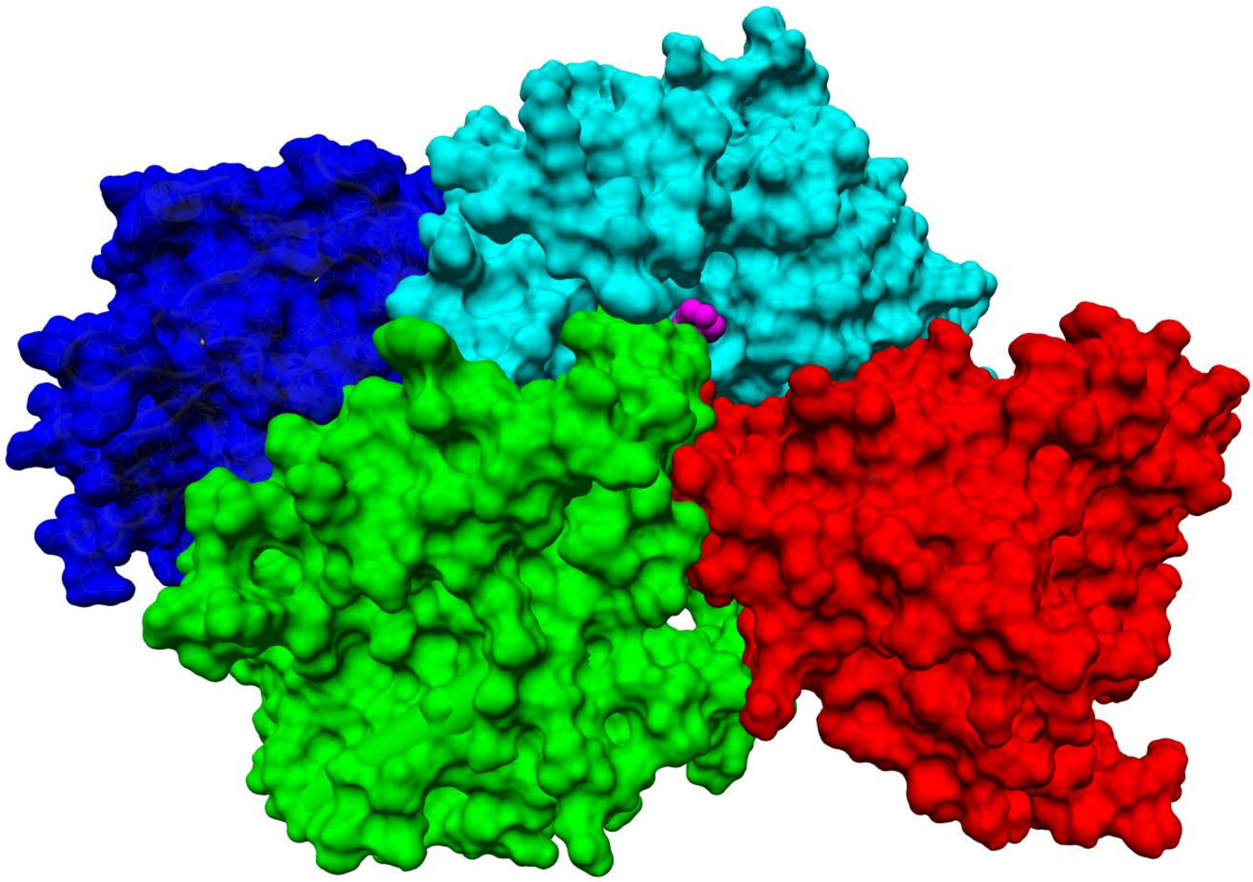

Figure S7. Proposed tetrameric structure of NitAb (monomers A red, B cyan, C green, D blue. Docked cinnamonitrile is shown in ball representation with magenta color.

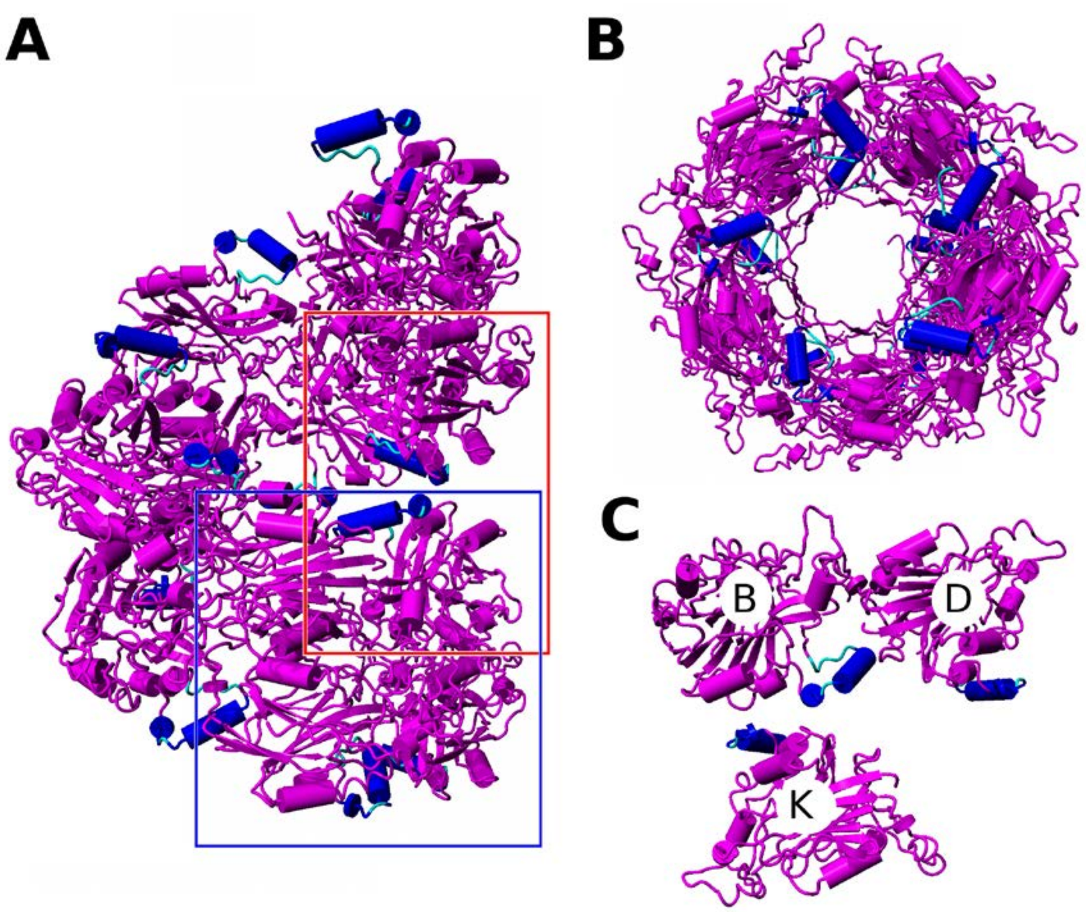

Figure S8. Models of NitAb. (A) Dodecamer (side view). The model is based on nitrilase NIT4 sequence, pdb code 6i00 [22], and represented by secondary structure cartoon style (side view). Loops HL1b (model 1: see Figure A1 in Appendix A) are colored blue. The red rectangular encloses monomers B, D and K, interaction surface of which forms D-interface (compare Appendix A) and was used for loop HL1b refinement. The blue rectangular determines the tetramer used for docking and molecular dynamics simulation (Figure S7). (B) Dodecamer (top view). (C) Refined model of HL1b loop. The model consists of monomers B, D and K, where loop HL1b of monomer B is sterically constrained by monomers D and K. See main text for reference.

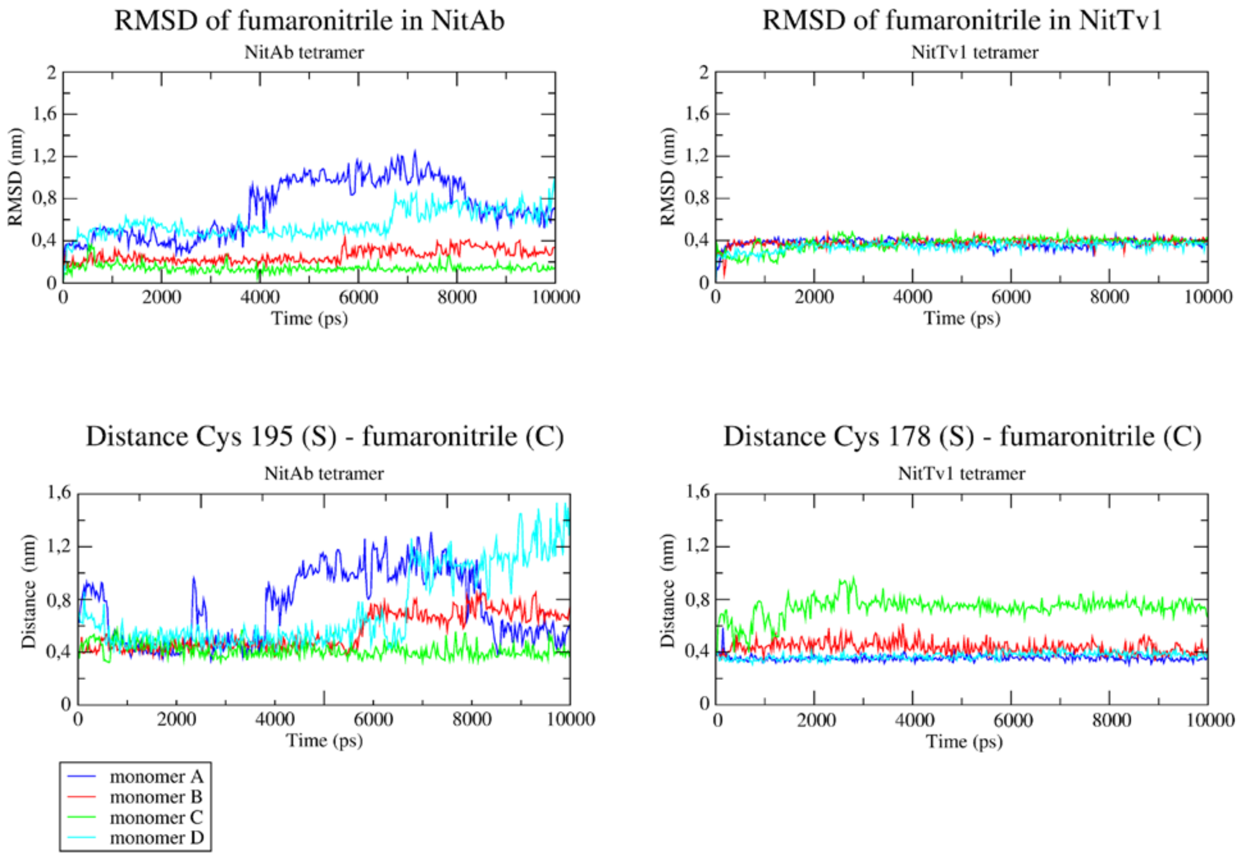

Figure S9. Plots of root means square deviations (RMSD) and distance between sulfur atom of catalytic C residue and cyano group carbon of fumaronitrile during molecular dynamics simulation of nitrilases NitAb (left column) and NitTv1 (right column).

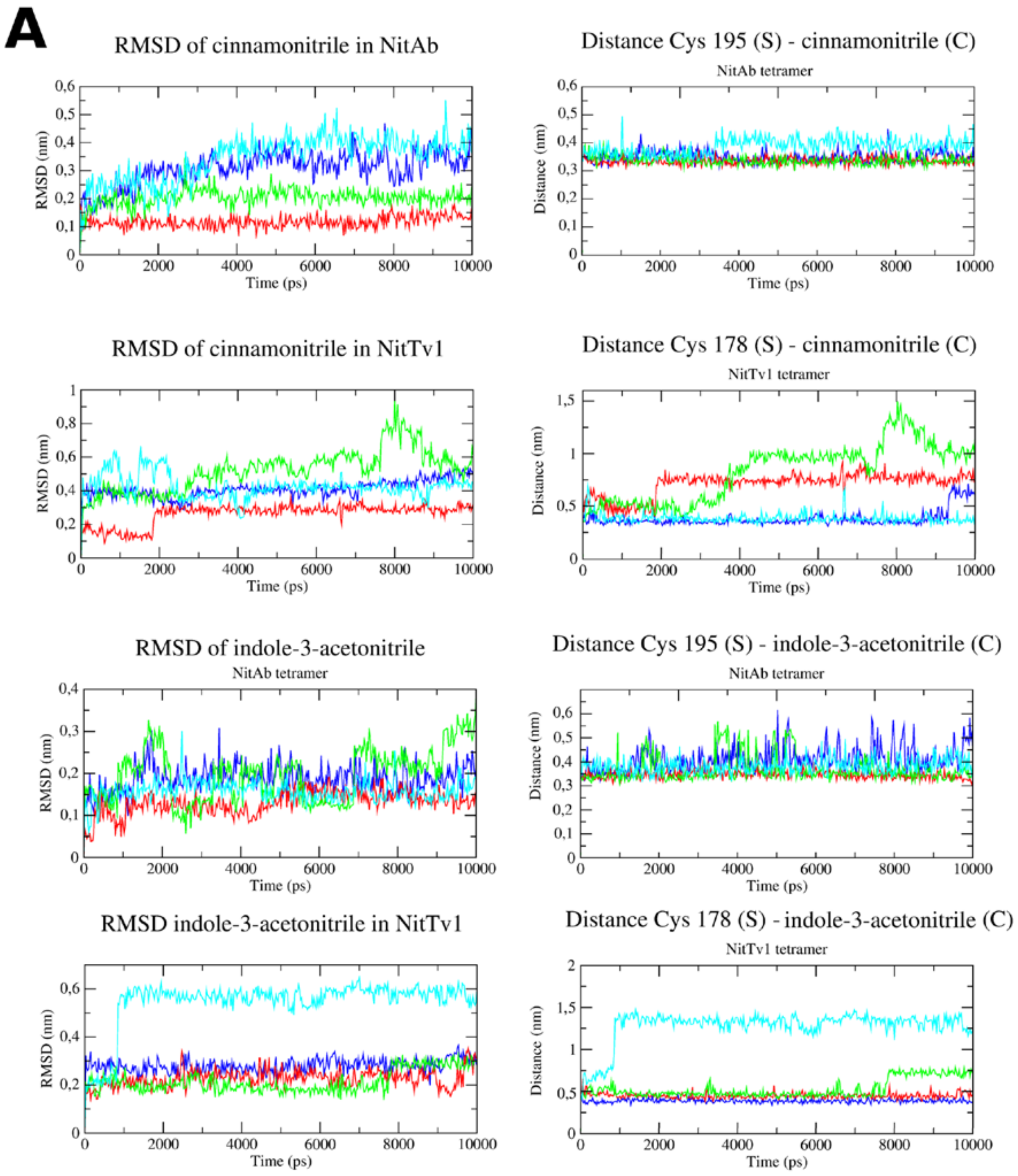

Figure S10 continues on next page.

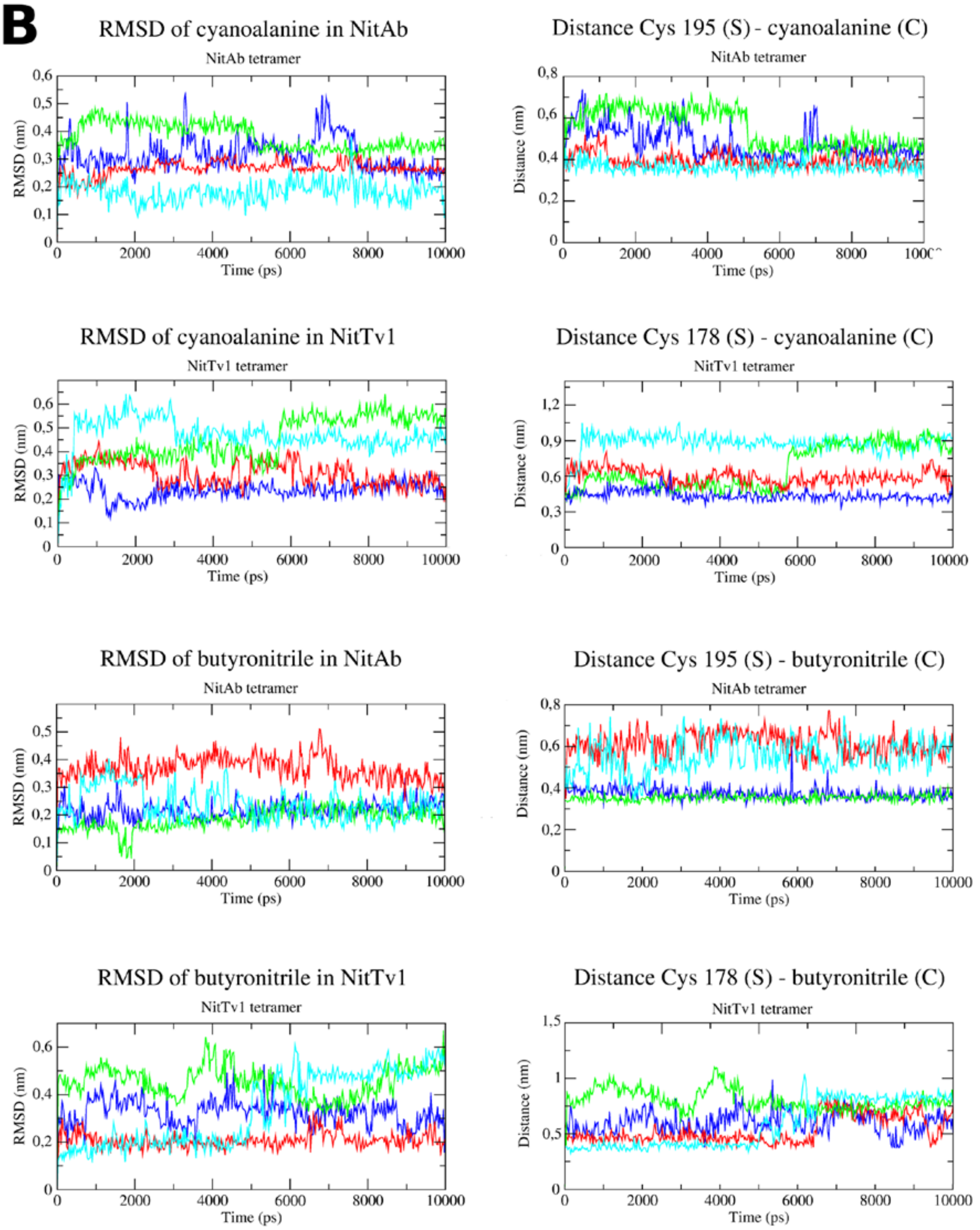

Figure S10. Root means square deviations (RMSD, left column) and distance between catalytic C (sulfur atom) and ligand (cyano group carbon) (right column) of selected (A) aromatic and (B) aliphatic ligands during molecular dynamics simulation.

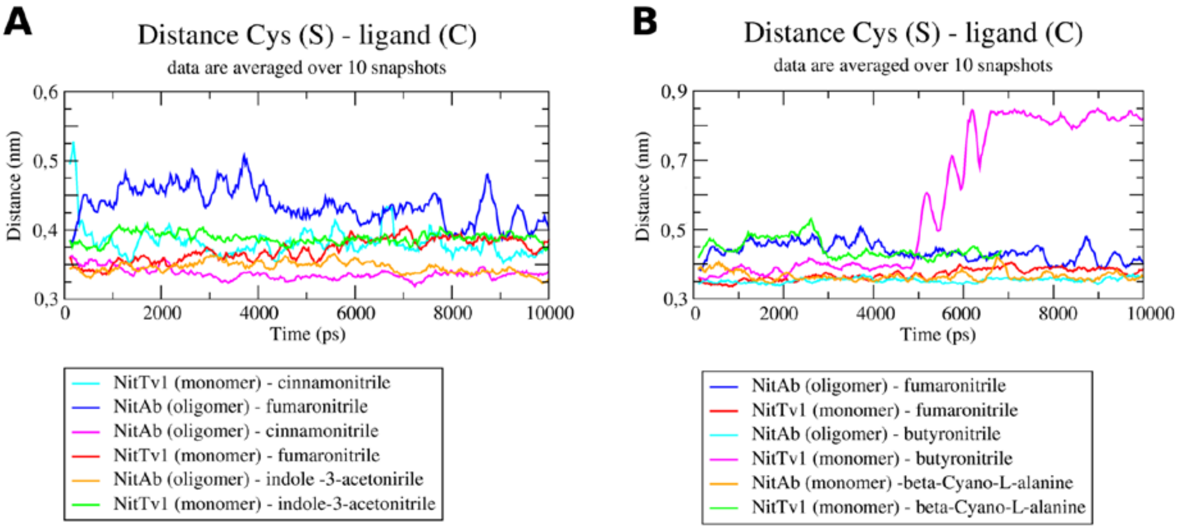

Figure S11. Comparison of representative distances between catalytic C (sulfur atom) and ligand (cyano group carbon) of selected (A) aromatic and (B) aliphatic ligands during molecular dynamics (MD) simulation. The representative distance was selected as the lowest distance in MD of the ligand in each complex.

Table S1 LC-MS analysis of the reaction mixtures from transformations of 3-phenylpropionitrile (PPN), cinnamonitrile (CN), phenylthioacetonitrile (PTAN) and 3-indoleacetonitrile (IAN)

Compound	RT (min)	MS-ESI *m/z* [1]
3-phenylpropionitrile	12.974	$[M+H]^+$ calcd for C_9H_9N 131.2, found 132
3-phenylpropionic acid	8.946	$[M+H]^+$ calcd for $C_9H_{11}O_2$ 151.2, found 151<br>$[M-H]^-$ calcd for $C_9H_9O_2$ 149.2, found 149
3-phenylpropionamide	3.553	$[M+H]^+$ calcd for $C_9H_{12}NO$ 150.2, found 150<br>$[M+H+CH_3CN]^+$ calcd for $C_{11}H_{15}N_2O$ 191.2, found 191
cinnamonitrile	20.214	$[M+CH_3COO]^-$ calcd for $C_{11}H_{13}NO_2$ 191.2, found 191
cinnamic acid	10.534	$[M+H+CH_3CN]^+$ calcd for $C_{11}H_{12}NO_2$ 190.2, found 190
cinnamamide	4.160	$[M+H+CH_3CN]^+$ calcd for $C_{11}H_{13}N_2O$ 189.2, found 189<br>$[M+H]^+$ calcd for $C_9H_{10}NO$ 148.2, found 148
phenylthioacetonitrile	17.513	$[M+H_2O]^+$ calcd for $C_8H_{10}NSO$ 168.2, found 168
phenylthioacetic acid	8.073	$[M-H]^-$ calcd for C_8H_7NOS 167.2, found 167;<br>$[M+HCOO]^-$ calcd for $C_9H_{11}NO_3S$ 213.2, found 213;
phenylthioacetoamide	3.824	$[M+H]^+$ calcd for $C_8H_{10}NOS$ 168.2, found 168;<br>$[M+H+CH_3CN]^+$ calcd for $C_{10}H_{13}N_2OS$ 209.2, found 209;<br>$[M-H]^-$ calcd for C_8H_8NOS 166.2, found 166
3-indoleacetonitrile	11.745	$[M+Cl]^-$ calcd for $C_{10}H_7N_2Cl$ 191.2, found 191
indole-3-acetic acid	5.300	$[M+Na]^+$ calcd for $C_{10}H_{11}ON_2Na$ 198.2, found 198
indole-3-acetamide	2.817	$[M+H]^+$ calcd for $C_{10}H_{11}ON_2$ 175.2, found 175;<br>$[M+H+CH_3CN]^+$ calcd for $C_{12}H_{14}ON_3$ 216.2, found 216

[1] MS-ESI *m*/*z* of the reaction products from fumaronitrile ((2*E*)-3-cyanoacrylic acid, (2*E*)-3-cyanoacrylamide) and 4-cyanopyridine (isonicotinic acid, isonicotinamide) were as described previously [18]. Data are shown for the products of NitTv1. NitAb gave the same products. The analytical method is described in the main text (section 4.8.).
